# intDesc-AbMut: A Tool for Describing and Understanding How Antibody Mutations Impact Their Environmental Interactions

**DOI:** 10.34133/csbj.0027

**Published:** 2026-04-27

**Authors:** Shuntaro Chiba, Masateru Ohta, Tsutomu Yamane, Yasushi Okuno, Mitsunori Ikeguchi

**Affiliations:** ^1^ HPC- and AI-driven Drug Development Platform Division, RIKEN Center for Computational Science, Yokohama 230-0045, Japan.; ^2^ Graduate School of Medicinal Life Science, Yokohama City University, Yokohama 230-0045, Japan.; ^3^ Department of Biomedical Data Intelligence, Graduate School of Medicine, Kyoto University, Kyoto 606-8507, Japan.

## Abstract

Structure-based antibody engineering requires systematic and reproducible characterization of residue-level interactions within antigen–antibody complexes. In our previous double-point mutation strategy, mutant selection was guided by preservation or enhancement of favorable local interactions; however, identifying and comparing these interactions depended on labor-intensive manual structural inspection. Here, we present intDesc-AbMut, a residue-centric computational tool that automates the extraction, classification, visualization, and descriptor generation of interactions involving designated residues in antigen–antibody complexes. The software explicitly defines 36 interaction types, encompassing conventional hydrogen bonds and van der Waals contacts as well as weak hydrogen bonds (e.g., CH···O and CH···π), sulfur-related contacts, bond dipole interactions, and orthogonal multipolar interactions. This fine-grained interaction taxonomy enables quantitative representation of local packing, weak hydrogen bonding, and directional electrostatic features surrounding mutated residues. As a proof of concept, we incorporate these interaction descriptors into a machine learning framework to evaluate whether they encode structural characteristics consistent with experimentally observed side-chain conformations. The results demonstrate that the descriptors capture meaningful structural signals underlying crystal-structure-like conformations. This analysis assesses descriptor informativeness rather than predicting binding affinity. In summary, intDesc-AbMut provides a systematic and extendable framework for mutation-focused interaction analysis in antibody structural studies and related protein design applications.

## Highlights

• intDesc-AbMut extracts and visualizes interactions around residues of interest
• The 36 defined interactions include weak and non-canonical types overlooked
• Interactions extracted by intDesc-AbMut can be converted into descriptors
• The descriptors can distinguish crystal-structure-like side-chain conformations
• intDesc-AbMut offers a framework that enables interaction-based mutation analysis

## Graphical Abstract

**Figure:**
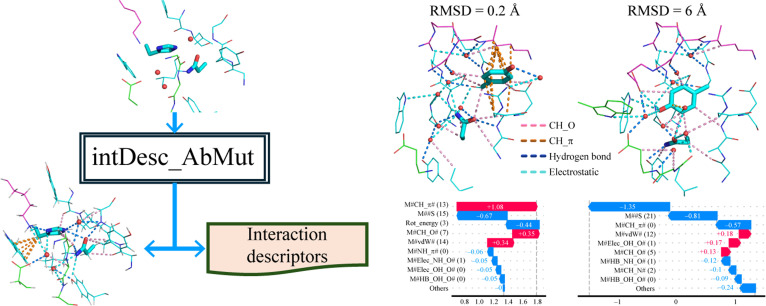

## Introduction

Antibody therapeutics represent a pivotal modality in the pharmaceutical industry, with considerable anticipation surrounding the development of novel antibody drugs [1]. Once a lead antibody is identified, structural modifications are continuously required to optimize its properties for pharmaceutical use. These modifications focus on improving activity, physicochemical properties, antigenicity-associated toxicity, and pharmacokinetic profiles.

One promising approach for structural antibody modification, single-point mutation (SPM), involves substituting a single amino acid in the antibody sequence to enhance its properties. SPM strategies utilize the 3-dimensional (3D) structures of antigen–antibody complexes [2–4], leveraging both experimentally determined and computationally modeled structures. However, although SPM-based designs have been widely explored, the impact of multiple simultaneous amino acid mutations—particularly those informed by 3D structural data—has been insufficiently investigated. This gap highlights the need for further methodological innovation in antibody engineering.

To broaden the scope of structural modification techniques, we previously introduced a double-point mutation (DPM) strategy that simultaneously mutates 2 spatially adjacent amino acids within an antibody. This strategy was successfully applied to generate multiple antibodies with improved activity [5,6]. Notably, when the 2 mutations were performed individually as SPMs, the resulting antibodies either showed markedly reduced activity or no expression at all. Thus, combining 2 beneficial SPMs may not necessarily achieve the same activity improvement as the DPM strategy.

When developing antibody drugs using the DPM strategy, many antigen/double-point mutant antibody (Ag/DPMAb) complex structures are generated via computational modeling. From this extensive pool of models, those expected to improve key physicochemical properties—such as antibody affinity and stability—must be selected. In one successful DPM application [6], we generated approximately 21,000 Ag/DPMAb complex structural models by introducing 19 types of mutations (excluding cysteine) at each of the 2 amino acids across 59 residue pairs. MM/GBVI energy [7] values were then used to filter structurally unfavorable models, particularly those exhibiting steric clashes between the 2 mutated residues. However, MM/GBVI energy was not applied to select antibodies for further experimental validation, as determining whether mutated side chains form favorable interactions is difficult when relying solely on electrostatic and van der Waals (vdW) terms, as these terms fail to capture the full spectrum of interactions, which also include weak hydrogen bonds (e.g., CH···π and CH···O interactions) [8], orthogonal multipole interactions [9,10], and S···O interactions [11]. Instead, 4 DPM antibodies were selected by prioritizing mutants with interactions around the mutated residues that were either preserved or enhanced relative to the wild-type (WT) antibody. These interactions were manually identified and analyzed using molecular graphics software. However, this method was time-consuming and introduced uncertainty as to whether all relevant interactions were accurately captured.

Therefore, the aim of this study is to overcome the limitations of manual interaction analysis by developing a software tool (intDesc-AbMut) that automatically identifies diverse interactions between mutant residues and their local environments from 3D structural data. To aid interpretation, intDesc-AbMut classifies and labels multiple interaction types, including not only commonly analyzed hydrogen bonds and vdW contacts but also weak hydrogen bonds, S···O, and orthogonal multipole interactions. Visual examination of these interactions is facilitated through molecular graphics. As an application, intDesc-AbMut can be used to analyze changes in interactions between WT and mutant antibodies in both SPM and DPM contexts.

Optionally, the extracted interactions can be converted into quantitative descriptors, allowing the local interaction state surrounding mutated residues to be systematically characterized. As an exploratory application of these descriptors, we construct a machine learning model to examine whether these descriptors capture structural features consistent with experimentally observed (“crystal-structure-like”) conformations. This proof-of-concept analysis evaluates the extent to which interaction descriptors encode structural plausibility of modeled side chains. Despite providing insights into descriptor informativeness, the model is not intended as a direct predictor of binding affinity or as a stand-alone tool for automated DPM candidate selection. By clarifying the structural information embedded in mutation-induced interaction patterns, this study establishes a systematic foundation for understanding and rationalizing antibody mutations at the residue–interaction level.

## Methods

### intDesc-AbMut

**Fig. 1.**
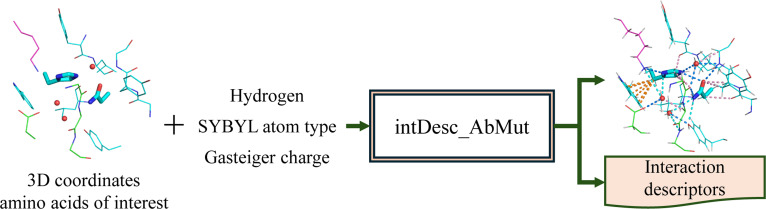
Overview of the intDesc-AbMut methodology.

intDesc-AbMut is a software tool designed to automatically extract interactions between a designated amino acid residue intended for mutation and its local environment from a 3D structure (Fig. 1). The environment considered by the tool includes (a) the remainder of the antibody, (b) the adjacent antigen, and (c) surrounding water molecules. For each interaction, intDesc-AbMut records the identities of interacting atom pairs and assigns an interaction-type label (e.g., hydrogen bond and CH···O). These labels enable the construction of interaction descriptors that enumerate the number of each interaction type present.

Interactions are primarily defined between heavy atoms, with some exceptions where interactions are defined between bonds. An interaction is assigned only when both the atom types and specific geometric constraints (e.g., distances and angles) are satisfied. Notably, intDesc-AbMut can automatically extract and label 36 different interaction types—including orthogonal multipole, S···O, and weak hydrogen bonds such as CH···O and CH···π interactions. Accordingly, each interaction type has an explicit operational definition. For every heavy atom in the mutant side chain, intDesc-AbMut evaluates the criteria for each interaction and outputs those that meet requirements.

#### Definitions of interactions

**Fig. 2.**
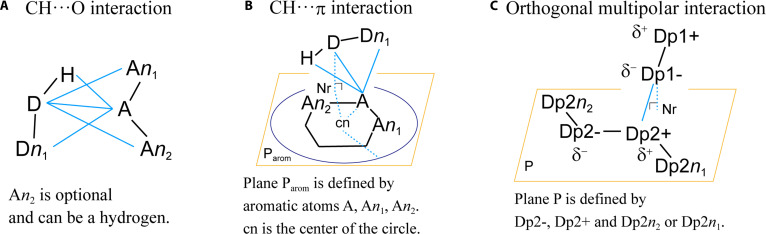
Interaction schemes of (A) CH···O, (B) CH···π, and (C) orthogonal multipolar interactions. D, heavy atom attached to donor hydrogen; H, hydrogen; Dn*_x_*, *x*th heavy atom covalently bonded to D; Dpn, heavy atom of dipole n; Dpn+, heavy atom of dipole n with a positive partial charge; Dpn−, heavy atom with a negative partial charge; black lines, any type of bond; cyan lines, distances between atoms belonging to 2 different residues; dashed cyan lines, auxiliary line for geometric reference; cn, center of the ring composed of aromatic atoms (the circle radius is set to *X* times the cn–A distance [parameter X, default = 1.4] used to evaluate the CH···π interaction); P, plane; P_arom_, plane formed by aromatic atoms; Nr, point where the perpendicular line from the atom to the plane P intersects with P.

Figure 2 illustrates the interaction schemes for CH···O, CH···π, and orthogonal multipolar interactions. The geometric conditions defining each interaction are summarized in Table 1. The full set of interaction definitions, including additional interaction types, is provided in Table S1.

**Table 1.** Definitions of CH···O, CH···π, and orthogonal multipolar interactions

Interaction	Label	Donor	D	A	Necessary conditions
CH···O	CH_O	CH	C	O	Dist. d(D:A) ≤ (RvdW(D) + RvdW(A) + 1.0 Å) Dist. d(D:A) ≤ d(Dn_1_:A) Dist. d(D:A) ≤ d(D:An_1_) Dist. d(D:A) ≤ d(D:An_2_) Dist. d(H:A) ≤ d(D:A) Dist. d(H:A) ≤ 3.22 Å Ang. 94.58° ≤ ∠(D:H:A) ≤ 180°
CH···π	CH_PI	CH	C	π	Dist. d(D:A) ≤ (RvdW(D) + RvdW(A) + 1.0 Å) Dist. d(D:A) ≤ d(Dn_1_:A) Dist. d(H:A) ≤ d(D:A) Dist. d(Nr:cn) ≤ d(cn:A)×1.4 (Dist. d(A:H) ≤ 3.195Å) OR ((Dist. 3.195 Å < d(A:H) ≤ 3.325 Å) AND (Ang. 124.455° ≤ ∠(D:H:A) ≤ 180.0°))
Orthogonal multipolar interaction	OMulPol				| δ^+^ − δ^−^ | ≥ 0.2 in dipole 1 | δ^+^ − δ^−^ | ≥ 0.2 in dipole 2 Dist. d(Dp1−:Dp2+) ≤ RvdW(Dp1−) + RvdW(Dp2+) + 0.7 Å Dist. d(Dp1−:Dp2+) ≤ d(Dp1+: Dp2+) Ang. 75° ≤ ∠(Dp2-:Dp2+:Dp1−) ≤ 105° Ang. 0° ≤ ∠(Nr:Dp1−:Dp2+) ≤ 35° Ang. 150° ≤ ∠(Nr:Dp1−:Dp1+) ≤ 180°

Label, the prefix assigned to each interaction; D, heavy atom attached to donor hydrogen; A, acceptor heavy atom; Dist., distance; Ang., angle; d(X:Y), distance between atoms X and Y; ∠(X:Y:Z), angle formed by atoms X, Y, and Z; RvdW(X), van der Waals radius of atom X

An interaction scheme specifies the motif required for each interaction, encompassing the required atoms, their bonding requirements, atom types (e.g., elements and hybridization), definitions of interatomic distances, bond angles, dihedral angles, planes, and, where applicable, constraints on atomic partial charges. In hydrogen-bond interactions, including weak hydrogen bonds, the acceptor heavy atom is denoted as A, the donor heavy atom bearing the donor hydrogen is denoted as D, and the donor hydrogen is denoted as H (Fig. 2A and B). Atoms covalently bound to D are labeled Dn_1_, Dn_2_, etc., whereas those adjacent to A are labeled An_1_, An_2_, etc.

For interactions defined between bond dipoles (Fig. 2C**)**, the interaction is specified by the atoms constituting each dipole, denoted Dpn+ and Dpn−. Here, the numeral following Dp indexes the dipole, and the subsequent “+” or “−” indicates the partial-charge sign of the atom. Likewise, δ^+^ and δ^−^ denote positive and negative partial charges, respectively. The antigen–antibody complex is provided as a MOL2 file that includes per-atom partial charges, which enables atom labeling based on Gasteiger-computed partial charges [12] recorded in the MOL2 file.

Table 1 details the necessary conditions for classifying each interaction type, where all listed conditions must be satisfied for assignment. Here, “necessary conditions” denote the geometric criteria (e.g., distance and angle thresholds) that must be satisfied for an interaction to be assigned. Constructed points, such as cn and Nr, may replace atom labels where appropriate. All distances and angles are computed from atomic coordinates provided in the MOL2 file. For dipole-involving cases—such as orthogonal multipolar interactions—the necessary conditions may also include atomic partial-charge specifications (e.g., δ^+^ and δ^−^).

Interaction labels comprise the interaction name or abbreviation and may include participating atom names. When visualized in PyMOL, these labels appear as objects and are written to a text file for programmatic aggregation. By tallying label entries, interaction descriptors can be constructed, such as counting the number of CH···O interactions.

Atom-type requirements for specific interactions (e.g., CH···O) are determined by SYBYL atom types listed in the MOL2 file [13], which encodes element and hybridization (e.g., C.3 for sp^3^ carbon). intDesc-AbMut determines whether an atom satisfies the element criteria for a given interaction by comparing the SYBYL type with the element specifications in the interaction definition.

Interactions between the mutated residue and its surrounding residues and water molecules are automatically extracted. Surrounding residues may belong to the antibody or antigen. Interactions within the mutated residue are not extracted, whereas those mediated by a single water molecule between the mutated residue and neighboring residues are extracted. Table 2 presents examples of interactions between a mutated residue (M), antibody (Ab), antigen (Ag), and water molecule (S) extracted by intDesc-AbMut.

**Table 2.** Interaction partners and their descriptor representations

Interaction partners	Descriptor representation
M–Ab	M#Label#Ab ^a^
M–Ag	M#Label#Ag
M–S	M#Label#S
M–M ^b^	M#Label#M
M–S–Ab	M#Label#S#Label#Ab
M–S–Ag	M#Label#S#Label#Ag
M–S–M ^b^	M#Label#S#Label#M

^a^ Label denotes the type of interaction label given in Table 1 and Table S1.

^b^ Interaction between mutated residues 1 and 2 for the DPM case.

### Preparing the input structure for intDesc-AbMut

#### Addition of hydrogen atoms

Antigen–antibody complex structures in Protein Data Bank (PDB) format typically lack hydrogen atoms required for interaction determination; thus, hydrogen addition is a required preprocessing step. Hydrogen addition is performed using the pdb2pqr 2.1.1 program [14,15].

#### Addition of atom-type and bond-type information

intDesc-AbMut relies on SYBYL atom and bond types [13], as well as partial charges to determine the types of interacting atoms. Additionally, the input must be a SYBYL MOL2 file with added hydrogens, atomic charges, atom types, and bond types. Accordingly, we developed pdb2mol2.py software to convert PDB files (with hydrogens) into MOL2 files that include all necessary information. The SYBYL atom type and SYBYL bond type required for input to intDesc-AbMut can be uniquely determined from the PDB-format amino acid residue name and PDB atom name. This conversion uses a dictionary that maps combinations of amino acid residues and atoms in the PDB file to appropriate SYBYL atom and bond types. The types of amino acids that can be handled by pdb2mol2.py are shown in Table S2. PDB files, which often contain many water molecules, are converted to MOL2 files by pdb2mol2.py.

As different hydrogen addition programs assign different atom names, distinct dictionary files were created for each program: pdb2pqr 2.1.1 [14,15] and the ff99SB-ildn force field [16,17] assigned by the pdb2gmx module of GROMACS 2021.4 [18]. The desired dictionary file can be specified when running pdb2mol2.py.

Atomic charges of each amino acid are also listed in the dictionary, with partial atomic charge information added to the MOL2 file accordingly. To calculate atomic partial charges, the N-terminus of each target amino acid was capped with an acetyl group and the C-terminus was capped with NMe, followed by hydrogen addition using the AmberTools tleap module [19]. Two disulfide-bonded cysteine residues were constructed by capping the N- and C-termini of each residue in a disulfide-bonded state. Amino acid structures were optimized using the AmberTools Sander module. Gasteiger charges were calculated using the Hgene program (Hydrogen Generation ENginE) [20].

### Files required for execution of intDesc-AbMut

In addition to the MOL2 file, intDesc-AbMut requires several supporting files: an interaction target molecule specification file that identifies the residues of interest, an interaction criteria file with interaction parameter definitions, a vdW radius definition file that defines the radius of each element, and an interaction priority file for label assignment when multiple interactions may exist between the same atoms.

### Example application of interaction descriptors generated by intDesc-AbMut

To demonstrate the application of interaction descriptors automatically extracted by intDesc-AbMut, we constructed a machine learning model that distinguishes whether each multiple side-chain conformation of specific amino acid residues in an antibody is similar to the crystal structure.

#### Preparing the dataset

A dataset of 520 antibody–protein antigen or antibody–peptide antigen complexes with good resolution (<2 Å) was obtained from SAbDab (2021 March 8) [21]; this database contains antibody structures from the PDB [22]. To exclude crystal lattice interactions, only complexes with a single asymmetric unit were retained. Structures with alternate residue conformations or incomplete side chains were excluded. Interactions cannot be defined when residues have incomplete structures, as their amino acid side chains cannot be determined from electron density. Therefore, we selected antigen–antibody complexes containing at least one fully determined residue located more than 4.5 Å from either a structurally incomplete residue or a crystallization agent (note that the crystallization agent was excluded from interaction extraction). This yielded 274 PDB entries and complex structures.

#### Selection of amino acid residues that generate side-chain rotamer structures

**Fig. 3.**
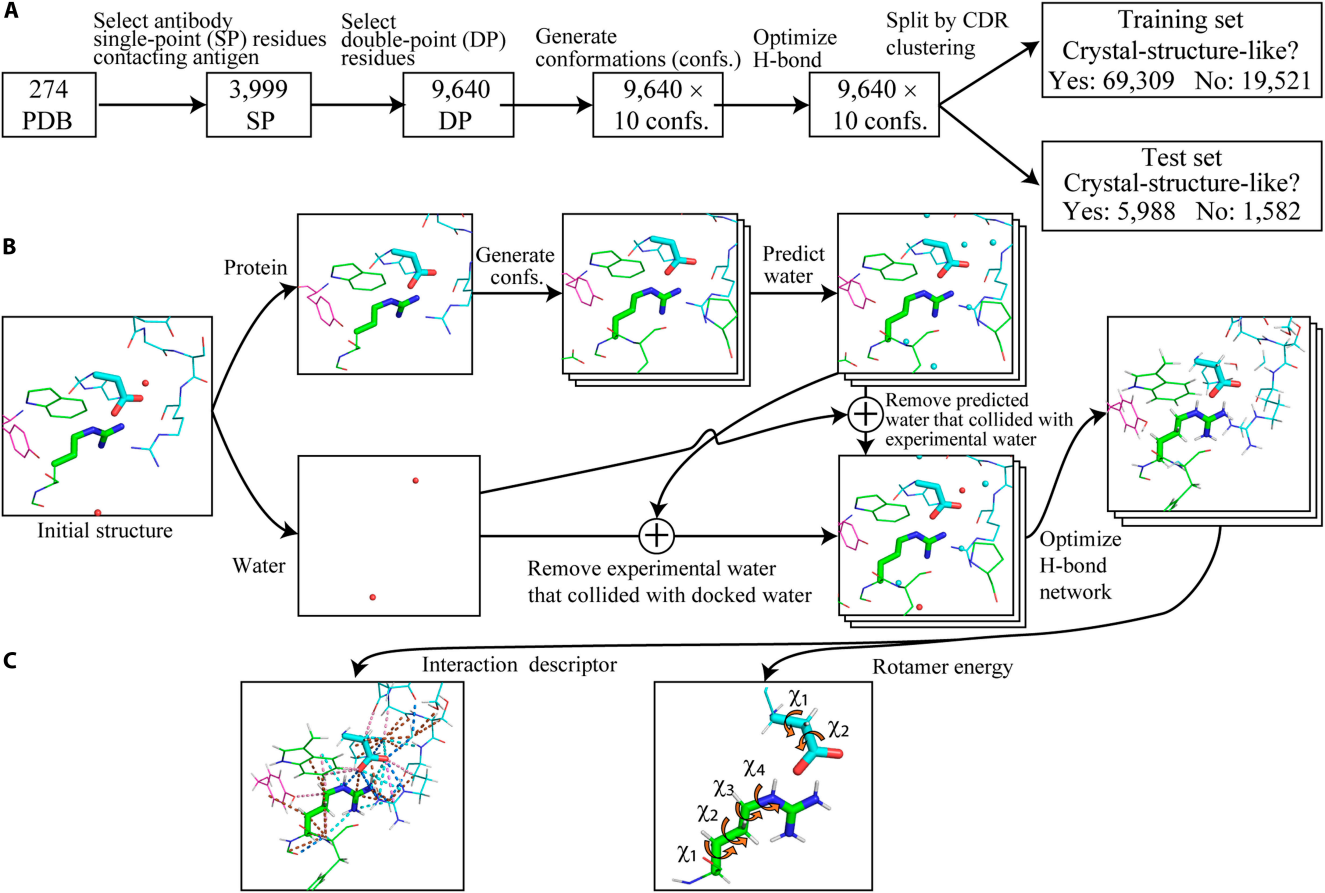
Preparation of training and test datasets. (A) Splitting the training and test sets. (B) Modeling of the side-chain structure and surrounding water. (C) Descriptor generation.

The machine learning model was used to distinguish whether the mutated side-chain structure resembled the crystal structure. To generate training data, we selected antibody residues at the antigen–antibody interface with a minimum heavy-atom distance to the antigen of less than 4.5 Å; glycine and alanine were excluded as they lack side-chain rotamers. Additionally, only residues with heavy atoms in the crystal structure located more than 4.5 Å from structurally incomplete residues or crystallization agents were used. These selected amino acid residues, named SPM residues, were used to select DPM residue pairs (Fig. 3A).

When the DPM strategy was adopted, we simultaneously mutated 2 adjacent residues at the antigen–antibody interface and generated a 3D model of the 2 residues. To allow the machine learning model to assess the likelihood of a DPM structure resembling a crystal structure, we designated a residue pair comprising an SPM residue and another residue located within 4.5 Å (minimum heavy-atom distance) as a DPM residue pair. Side-chain structures for each DPM residue pair were constructed by combining the 2 residues (Fig. 3A). Again, glycine and alanine were excluded from DPM residue pairs.

#### Generation of side-chain structures

For the DPM residue pair, multiple side-chain structures were generated by mutating residues to the same amino acid identity. Protein and experimental water molecules were separated, and 10 conformations were generated for each mutated residue using FoldX5 [23] (Fig. 3B). Water molecules were docked onto each side-chain structure using FoldX4. Experimental water molecules that collided with the docked water molecules (threshold: 1.5 Å inter-heavy-atom distance) were deleted; those that did not were returned to their original positions. Hydrogens were then added, and the hydrogen-bond network was optimized using pdb2pqr 2.1.1 [14,15].

#### Determining the crystal-structure-likeness of side-chain structures

For each generated side-chain structure, the root mean square deviation (RMSD) between the model and crystal structure side chain (excluding $C_{\beta }$) was calculated using side-chain heavy-atom coordinates with the DockRMSD program [24]. Structures with RMSD < 1 Å were labeled “crystal-structure-like”, whereas those with RMSD ≥ 1 Å were labeled “non-crystal-structure-like”. The RMSD calculations also accounted for symmetry, such as benzene ring rotation [24].

#### Selecting the training and test sets

The training and test sets were selected from side-chain structure data to maximize antibody diversity, based on complementarity-determining region (CDR) amino acid sequences. First, clustering was performed based on the amino acid sequences of CDRs for the 274 complex structures (Figs. S1 and S2); the CDR residue numbers were standardized by the IMGT (International ImMunoGeneTics information system) numbering scheme [25] using ANARCI (Antigeneric Numbering And Receptor Classification Integration) [26] and HMMER (version 3.3.1) [27], then clustered into 51 groups using single-linkage clustering in SciPy 1.5.3 [28] (Figs. S1 and S2). Maximum and average CDR identities between cluster members from different groups were 40% and 34% ± 4.9%, respectively. The 51 clusters were randomly split 2:1 into 34 clusters (246 PDB) for the training set and 17 clusters (28 PDB) for the test set. PDB IDs and cluster IDs are provided in Table S3. The number of “crystal-structure-like” and “non-crystal-structure-like” data for training and test sets are shown in Fig. 3A.

#### Generation and aggregation of interaction descriptors

intDesc-AbMut was applied to all antigen–antibody complex structures in our dataset (Fig. 3A) to extract interactions. Each extracted interaction included the interacting partner and interaction type, such as M#CH_O#Ab (between mutated residue and antibody residue) or M#CH_O#Ag (between mutated residue and antigen residue). After extraction, interactions were grouped by type rather than by individual partners before counting to avoid inflating descriptor counts. For example, in the case of CH···O interactions, M#CH_O#Ab, M#CH_O#Ag, etc. were collectively designated as M#CH_O# interactions (CH···O interaction including mutated residue, regardless of the interacting partner) and then totaled. Interactions mediated by water were also grouped and counted as a single descriptor. For example, as the number of possible interaction combinations for M#interaction1#S#interaction2#Ab is relatively large, rather than counting each interaction combination, we grouped these combinations as an interaction of mutant residues mediated by water, M##S##, which was then treated as a single descriptor. Similarly, interactions between water molecules and the mutated residue were grouped and counted as a single descriptor. For example, interactions such as M#interaction1#S were grouped as water–mutant interactions (M##S) instead of being counted individually. The 5 metal interactions and 3 ion interactions listed in Table S1 were excluded from the training data, resulting in 30 interaction descriptors (Table S4).

#### Calculation of side-chain rotamer energies

Side-chain structures with high dihedral-angle energies are rare. Additionally, the interaction descriptor is a measure of the goodness of the interaction between the mutated residue and its surroundings, not a direct evaluation of the poorness of the side-chain structure. Hence, we developed a rotamer energy calculation program (rotamer_frequency.py) to apply rotamer energy as a descriptor of structural “poorness”, calculated using the following equation:

$$\text{Energy}=-\mathrm{RT}\ln\left(\text{probability}/\text{maximum probability}\right)$$ (1)

where *R* and *T* represent the gas constant [0.001987 kcal/(mol K)] and a temperature of 300 K, respectively.

Rotamer probabilities for residue conformations were obtained from a backbone-dependent rotamer library that compiles the relationships between rotamer conformations and the frequencies of experimental structures [29]. The maximum value of each rotamer probability for a side chain was selected from the probability values defined for the same backbone geometry. The rotamer probabilities of the 2 amino acid residues constituting DPM, which were assumed to be independent, were calculated by multiplying the existence probabilities of each residue.

#### Model training

We constructed a machine learning model using 30 interaction descriptors to classify whether a side-chain structure is “crystal-structure-like” (RMSD < 1 Å). The training set comprised 34 CDR clusters. XGBoost 1.5.1 [30] was used as the machine learning method, with hyperparameters optimized by grid search via Scikit-Learn 1.5.0 [31] (Table S5A). The group shuffle partitioning method was applied, using 64% of clusters for model building, 16% for model performance monitoring (early stopping), and 20% for model evaluation. The CDR cluster groups defined in the “Selecting the training and test sets” section were used to divide the group units. Boosting rounds were capped at 20,000, with early stopping applied if no improvement in the Matthews correlation coefficient (MCC) [32] was observed after 100 consecutive rounds. The learning rate was set to 0.025. Grid search was repeated 200 times, then the hyperparameter that maximized the average MCC was selected. With the selected hyperparameters, 80% of clusters were randomly used for training and 20% were used for early stopping (Table S5B). A second machine learning model was created using 31 descriptors: 30 interaction descriptors plus the rotamer-energy descriptor (Table S5B).

#### Contribution of each descriptor in the machine learning model

We used the permutation importance method to evaluate the contribution of each descriptor to improving the accuracy of the machine learning model [33]. The MCC calculated on the test set using all descriptors was defined as the baseline performance. Subsequently, the values for each descriptor were randomly shuffled, and the MCC was recalculated 100 times. The mean reduction from the baseline MCC was used as the criterion.

#### Contribution of each descriptor to crystal-structure-likeness classification

Finally, we assessed model ability to distinguish crystal-structure-likeness based on the values of each descriptor used as inputs for a given side-chain structure. Specifically, we used SHapley Additive exPlanations values [34] (https://github.com/shap/), which are based on game theory, to quantify the additive contribution of each descriptor to model prediction performance.

## Results and Discussion

### Novel contribution of intDesc-AbMut

Weak and noncanonical interactions such as CH···O and CH···N hydrogen bonds, CH···π interactions, sulfur-involving contacts (e.g., S···O, S···N, S···S, and SH···π), and dipole-based directional interactions are increasingly recognized as important contributors to molecular recognition and structural stabilization in biological systems. CH-based hydrogen bonds and associated weak hydrogen bonds have been extensively reviewed in structural chemistry and biomolecular contexts [8,35]. CH···π interactions are well-documented stabilizing forces in proteins and protein–ligand complexes [36]. Sulfur-involving interactions, including chalcogen bonding and sulfur–aromatic contacts, are structurally relevant but often underappreciated contributors [37,38]. More generally, higher-order multipole interactions provide a theoretical basis for directional dipole-mediated contacts [39].

However, these interaction subtypes are not uniformly extracted across existing tools. Widely used programs—including ProLIF, BINANA, Arpeggio, PLIP, GetContacts, PoseView, HBPLUS, and PyContact—have substantially advanced the structural analysis of biomolecular complexes; however, these tools only support the automated detection of common interactions such as hydrogen bonds, salt bridges, π–π stacking, cation–π interactions, hydrophobic contacts, and metal coordination. As summarized in Table 3, intDesc-AbMut not only supports conventional interaction categories but also extends explicit classification to a broader spectrum of weak and sulfur-related contacts. In particular, intDesc-AbMut explicitly defines interaction subtypes such as CH···O, CH···π, CH···N, CH···S, OH···S, NH···S, SH···O, SH···N, SH···S, OH···π, SH···π, S···O, S···N, and S···S using defined geometric and atom-type criteria. The software further detects orthogonal multipolar and bond dipole interactions, which are rarely included in general-purpose interaction profilers. Moreover, hydrogen bonds and longer-range electrostatic interactions are distinguished using distance-based thresholds, enabling more granular categorization of polar contacts.

**Table 3.** Interaction types supported by intDesc-AbMut and other representative biomolecular interaction analysis tools. Check marks (✓) indicate that the corresponding interaction category is supported by each tool. Halogen-related interactions are not included in the table. Rightmost columns list the interaction types explicitly defined in intDesc-AbMut and their labels. References for comparison software: ProLIF [49], BINANA [50], Arpeggio [51], PLIP [52], GetContacts [53], PoseView [54], HBPLUS [55], and PyContact [56].

Interaction	ProLIF	BINANA	Arpeggio	PLIP	GetContacts	PoseView	HBPLUS	PyContact	intDesc interaction	intDesc label
Hydrogen bond	✓	✓	✓	✓	✓	✓	✓	✓	Hydrogen bond (<3.2 Å)	HB_OH_O; HB_NH_O; HB_OH_N; HB_NH_N
Electrostatic interaction	✓								Electrostatic (≥3.2 Å)	Ele_OH_O; Ele_NH_O; Ele_OH_N; Ele_NH_N
Salt bridge		✓	✓	✓	✓			✓	Hydrogen bond or electrostatic	
Polar contact			✓							
Weak hydrogen bond			✓							
Carbonyl interaction			✓							
π–π stacking (face-to-face)	✓	✓	✓	✓	✓				π–π stacking	PI_PI
π–π stacking (edge-to-face)	✓	✓	✓	✓	✓				CH···π	CH_PI
Cation–π	✓	✓	✓	✓	✓				NH···π	NH_PI
Amide–π			✓				✓		NH···π	NH_PI
Amide–amide			✓							
Sulfur–aromatic			✓						S···π	S_PI
									CH···O	CH_O
									CH···N	CH_N
									CH···S	CH_S
									OH···S	OH_S
									NH···S	NH_S
									SH···O	SH_O
									SH···N	SH_N
									SH···S	SH_S
									OH···π	OH_PI
									SH···π	SH_PI
									S···O	S_O
									S···N	S_N
									S···S	S_S
									Orthogonal multipole interaction	OMulPol
									Bond dipole	Dipo
Hydrophobic contact	✓	✓	✓	✓		✓		✓	van der Waals	vdW
van der Waals		✓	✓		✓		✓		van der Waals	vdW
Steric clash		✓	✓							
Metal coordination	✓	✓	✓			✓			Metal	Fe_A(element); Zn_A(element); Ca_A(element); Mg_A(element); Ni_A(element)
									Ion	Na_A(element); K_A(element); Cl_A(element)
Water bridge				✓	✓				Water-mediated	M#Label#S; M#Label#S#Label#Ab; M#Label#S#Label#Ag; M#Label#S#Label#M (M: Mutant residues to be analyzed, S: Solvent(water), Ab: Antibody, Ag: Antigen)

### Example of interaction visualization using intDesc-AbMut

As an illustrative example of intDesc-AbMut, we examined the single-point mutant H-N57Y in the Tissue Factor–anti-Tissue Factor antibody complex, where Asn57 in the heavy chain of crystal structure [40] is mutated to Tyr. This mutation increases the binding affinity of the anti-Tissue Factor antibody 3.1-fold [6]. The WT structure was generated by adding hydrogens to the crystal structure (PDB ID: 1JPS) using pdb2pqr; this was followed by conversion to MOL2 file format using pdb2mol2.py. The mutant H57Y model was constructed in our previous study [6], where hydrogens were added to the crystal structure (1JPS) using Protonate3D [41] in the Molecular Operating Environment [42], and the structure was optimized using the ff14SB molecular force field. Mutant structures were created using the Molecular Operating Environment residue scan module, outputted as PDB files, then converted to MOL2 files using pdb2mol2.py.

#### WT (N57) interactions

**Fig. 4.**
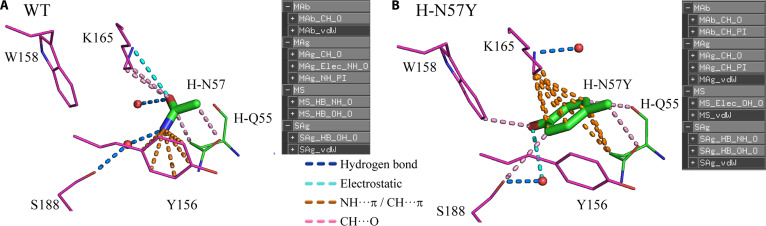
Changes in interactions caused by the H-N57Y single-point mutation. (A) Wild-type and (B) single-point mutant H-N57Y. Hydrogen bonds are shown in blue, electrostatic interactions are shown in cyan, NH···π and CH···π interactions are shown in orange, and CH···O interactions are shown in pink. van der Waals interactions are not shown. Green indicates antibody, and magenta indicates antigen. Hydrogens are not shown. Only the side-chain structures of N57 and N57Y are shown (sticks). Only the oxygen atoms of water molecules are shown (red spheres). W158 in (A) does not interact with the side chain but is shown for comparison with (B).

After obtaining MOL2 files for the WT and mutant, intDesc-AbMut was employed to automatically determine interactions between residue 57 and its surroundings in both structures, producing a PyMOL Script file (pml file) for visualization. Interactions were clearly visualized by loading the corresponding MOL2 file as the input for intDesc-AbMut into PyMOL [43], then loading the pml file (Fig. 4).

Analysis of the WT heavy chain Asn57 (H-N57) side chain with intDesc-AbMut revealed numerous NH···π interactions with Y156 (Fig. 4A). In this interaction, the hydrogen atom of NH acts as a donor whereas the π electrons of the aromatic ring act as an acceptor. The interaction between the NH_2_ group and S188 was mediated by water through hydrogen bonds. The carbonyl oxygen of the H-N57 side chain, which acted as an acceptor, interacted with the $N_{\zeta }$H_3_ of the K165 side chain, which acted as a donor. Electrostatic interaction, MAg_Elec_NH_O, was assigned instead of a hydrogen bond because the distance between N and O was greater than 3.2 Å. intDesc-AbMut distinguishes hydrogen bonds (≤3.2 Å) and electrostatic interactions (>3.2 Å) based on interatomic distances, and visualizes hydrogen-bond distances with color changes. Therefore, in intDesc-AbMut, electrostatic interactions may effectively be interpreted as long-range hydrogen bonds. Notably, salt bridges are not explicitly classified; instead, they are categorized as either hydrogen bonds or electrostatic interactions according to the heavy-atom distance. The carbonyl oxygen of the H-N57 side chain also formed CH···O interactions with $C_{\delta }$H_2_ and $C_{\varepsilon }$H_2_ of the K165 side chain and $C_{\gamma }$H_2_ of the antibody H-Q55 side chain, indicating weak hydrogen bonds, in which the hydrogen of CH acted as a donor and O acted as an acceptor. Thus, carbonyl oxygen recognition of the H-N57 side chain was achieved through electrostatic interactions with the $N_{\zeta }$H_3_ of K165 and through weak hydrogen bonding with the CH_2_ side chain. Additionally, $C_{\beta }$H_2_ of the H-N57 side chain interacted with carbonyl oxygen of the antibody H-Q55 side chain via CH···O.

#### N57Y mutant interactions

intDesc-AbMut revealed the loss of 2 interactions between the WT (H-N57) and single-point mutant (H-N57Y): the NH···π interaction with Y156 and the CH···O interaction between $C_{\delta }$H_2_ and $C_{\varepsilon }$H_2_ of the K165 side chain and the carbonyl oxygen of the N57 side chain (Fig. 4B). However, the phenol ring of the H-N57Y side chain, introduced by mutation, formed new CH···π interactions with the $C_{\delta }$H_2_ and $C_{\varepsilon }$H_2_ of the K165 side chain and the $C_{\gamma }$H_2_ of the antibody H-Q55 side chain, surpassing the lost interactions. The water-mediated interaction with S188 shifted from NH2 in N57 to phenol OH in N57Y, and a CH···O interaction between the N57Y side chain $C_{\varepsilon }$H and the S188 side chain was added. W158 of the antigen, previously noninteracting, formed a CH···O interaction with the side-chain indole ring $C_{\eta }$H as the donor and the phenolic oxygen atom of N57Y as the acceptor. These interaction changes accounted for the increased activity of H-N57Y, consistent with previous studies [6].

#### WT (N34/H91) interactions

A second example involved intDesc-AbMut analysis of the L-N34D/H91S mutant, which exhibited increased binding affinity from 45 to 25 pM. The WT structure was generated using methods similar to those for the H-N57Y mutant. The L-N34D/H91S mutant structure was similarly created based on the 1JPS crystal structure using the Molecular Operating Environment residue scan module.

**Fig. 5.**
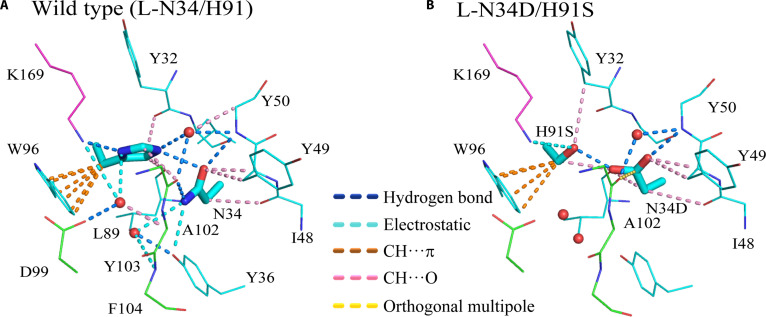
Changes in interactions caused by the L-N34D/H91S double-point mutation. (A) Wild-type and (B) double-point mutant L-N34D/H91S. Hydrogen bonds are shown in blue, electrostatic interactions are shown in cyan, CH···π interactions are shown in orange, CH···O interactions are shown in pink, and orthogonal multipole interactions are shown in yellow. van der Waals interactions are not shown. Green indicates antibody heavy chain, cyan indicates antibody light chain, and magenta indicates antigen. Hydrogens are not shown. Only the side-chain structures of N34, N34D, H91, and H91S are shown (sticks). Only the oxygen atoms of water molecules are shown (red spheres). Y36, L89, D99, or F104 does not interact with the side chain in (B) but is shown for comparison with (A).

In the WT, a network of hydrogen bonds formed between the antigen K169 and the antibody H91, between H91 and N34, and between N34 and Y50 (Fig. 5A). This structure was reinforced by CH···π interactions between the C_β_ of H91 and W96, CH···O interactions between the C_δ_H of H91 and both L89 and Y32, and CH···O interactions between the O_δ_ of N34 and Y49. The N_ε_H of H91 formed a hydrogen bond with water, facilitating additional hydrogen-bond and CH···O interactions with Y50.

#### N34D/H91S mutant interactions

For the L-N34D/H91S mutant, the K169–H91S interaction remained present but was reclassified as electrostatic owing to a distance of 3.9 Å (Fig. 5B). Additionally, hydrogen bonds formed between H91S and N34D, and between N34D and Y50, creating an interaction network similar to that of the WT. This network was further reinforced by CH···π interactions between the C_β_ of H91S and W96, CH···O interactions between the C_γ_ of H91S and Y32, CH···O interactions between the C_β_ of H91S and one of the O_δ_ atoms of N34D, and CH···O interactions between the other O_δ_ atom of N34D and Y49. The backbone carbonyl oxygen of L89 formed a CH···O interaction with the C_δ_H of H91 in the WT, but formed a CH···O interaction with the C_β_H of N34D in the L-N34D/H91S mutant. Additionally, an orthogonal multipolar interaction between the backbone carbonyl oxygen of the antibody heavy chain A102 and the C_γ_ of N34D was detected in the L-N34D/H91S mutant but was absent in the WT. Orthogonal multipolar interactions occur when the δ^−^ of a dipole, such as C=O, interacts perpendicularly with the δ^+^ of another dipole. Overall, intDesc-AbMut demonstrated that interactions in the WT were either preserved or enhanced in the L-N34D/H91S mutant, supporting its selection for experimental validation.

The ability of intDesc-AbMut to visually and easily define the types and numbers of interactions between specific residues and their surroundings in both WT and mutant structures has substantial value for understanding mutation effects.

### Hydrogen addition

As defined in Table S1, interaction extraction in intDesc-AbMut utilizes SYBYL atom types (to determine whether atoms satisfy interaction definitions such as donor or acceptor), geometric parameters including interatomic distances and angles, and atomic partial charges required for certain interaction criteria. Because hydrogen atoms are explicitly included in these definitions, hydrogen addition has a substantial impact on the results of intDesc-AbMut. For example, the SYBYL atom types assigned to oxygen atoms differ between –COOH and –COO^−^ groups, which in turn affects donor/acceptor assignment. This also influences atomic partial charges calculated using the Gasteiger method. In addition, for molecules—particularly water molecules—the coordinates of the added hydrogen atoms can markedly affect interaction detection. In this study, hydrogen atoms were added using pdb2pqr to maintain consistent structural quality. However, more rigorous analyses should consider hydrogen addition methods that consider protonation states, tautomeric forms, and optimization of hydrogen-bond networks.

### Interaction descriptor generation

Applying intDesc-AbMut to antigen–antibody complex structures can reveal the heavy-atom-level interaction patterns around specified residues. The extracted interactions are compiled into a list, which can be used to perform various statistical analyses.

As an example, we present an interaction analysis of antigen–antibody interface residues derived from the crystal structures of antigen–antibody complexes. The crystal structures that served as the initial structure for constructing the machine learning model described below were used for the analysis. This analysis includes interactions between antibody side chains and between antibody and antigen side chains, but does not include interactions between antibody side chains and water molecules. To analyze the interaction frequencies in crystal structures, we quantified the interactions identified by intDesc-AbMut for crystal structures corresponding to 9,640 double-point pairs (Fig. 3A). Prior to descriptor calculation, pdb2pqr was applied to the crystal structures under the same conditions used during dataset construction. Interaction descriptors were then computed with intDesc-AbMut, and the counts of each interaction type were summarized.

**Fig. 6.**
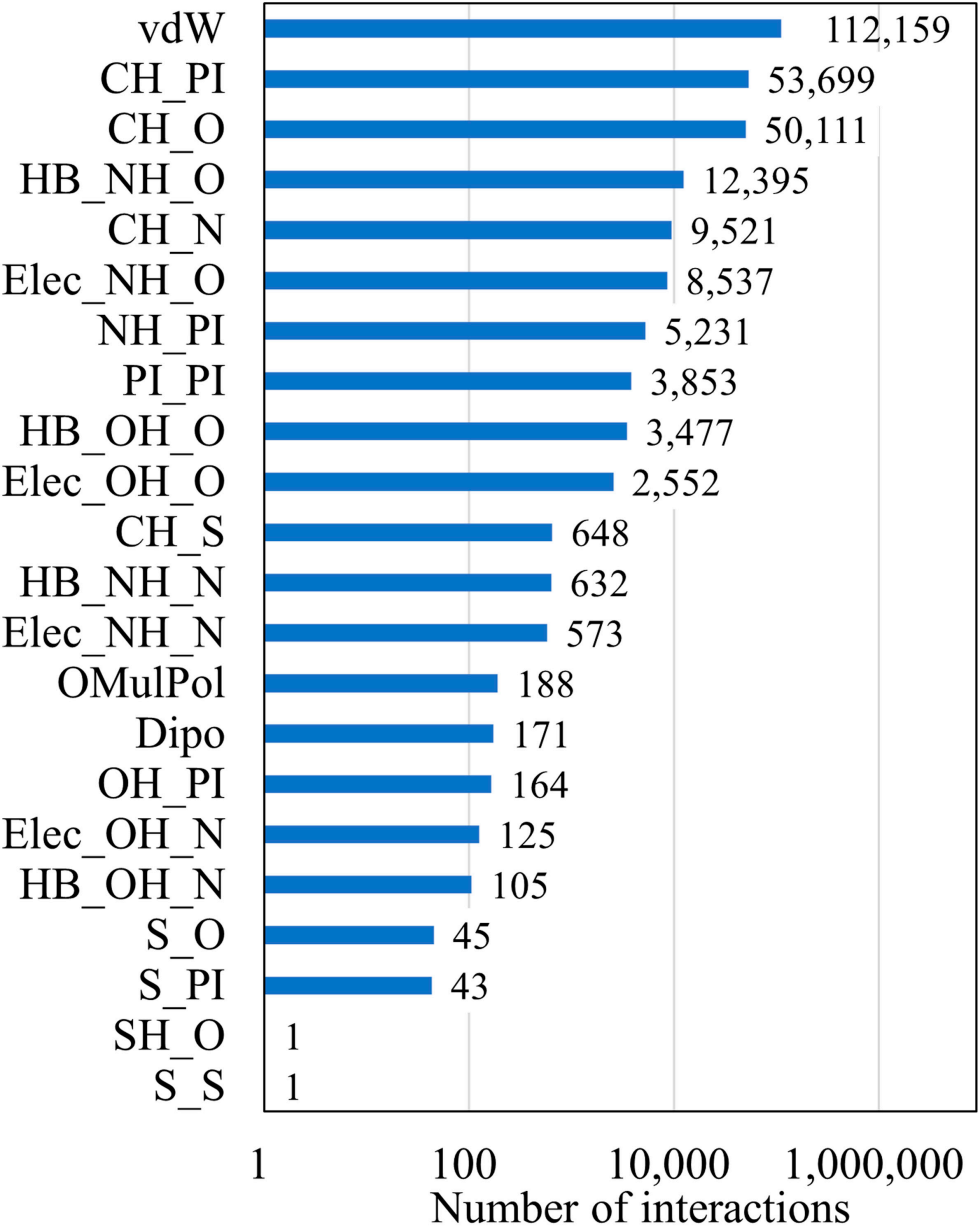
Frequency of different interaction types based on the crystallographic structures of side chains in the crystal-structure-likeness dataset of antigen–antibody complexes. Interaction labels are defined in the intDesc label field in Table S1. Undetected interactions are not shown.

Figure 6 shows the frequency of each interaction type, calculated by compiling the lists of interactions for each analyzed residue and aggregating them by interaction label. Among the residues at the antigen–antibody interface in the analyzed crystal structures, as well as residues that were 3-dimensionally adjacent to the interface residues, vdW interactions were the most prevalent interactions with surrounding atoms (excluding water), followed by weak hydrogen-bond interactions such as CH···π (CH_PI) and CH···O (CH_O) interactions. We also observed interactions specific to intDesc-AbMut, including orthogonal multipolar interactions, bond dipole interactions, and S···O interactions (S_O), although at relatively low frequencies.

The computation time of intDesc-AbMut, including descriptor generation from the input structure and PyMOL script creation, was 5.3 ± 1.6 s (mean ± standard deviation) per double-point pair, with a median of 5.0 s. The reported time corresponds to execution using a single CPU core of an Intel Xeon Max 9480 processor.

### Proof-of-concept evaluation of descriptor informativeness

To assess whether the constructed interaction descriptors contain structural information capable of explaining experimentally determined structures, we performed a proof-of-concept machine learning analysis. According to the procedure described in the “Example application of interaction descriptors generated by intDesc-AbMut” section, both high-resolution antigen–antibody complex crystal structures and side-chain model structures generated from these complexes were classified as either “crystal-structure-like” or “non-crystal-structure-like” based on their RMSD values relative to the crystal structures. We then examined whether the interaction descriptors were capable of distinguishing these classes.

**Fig. 7.**
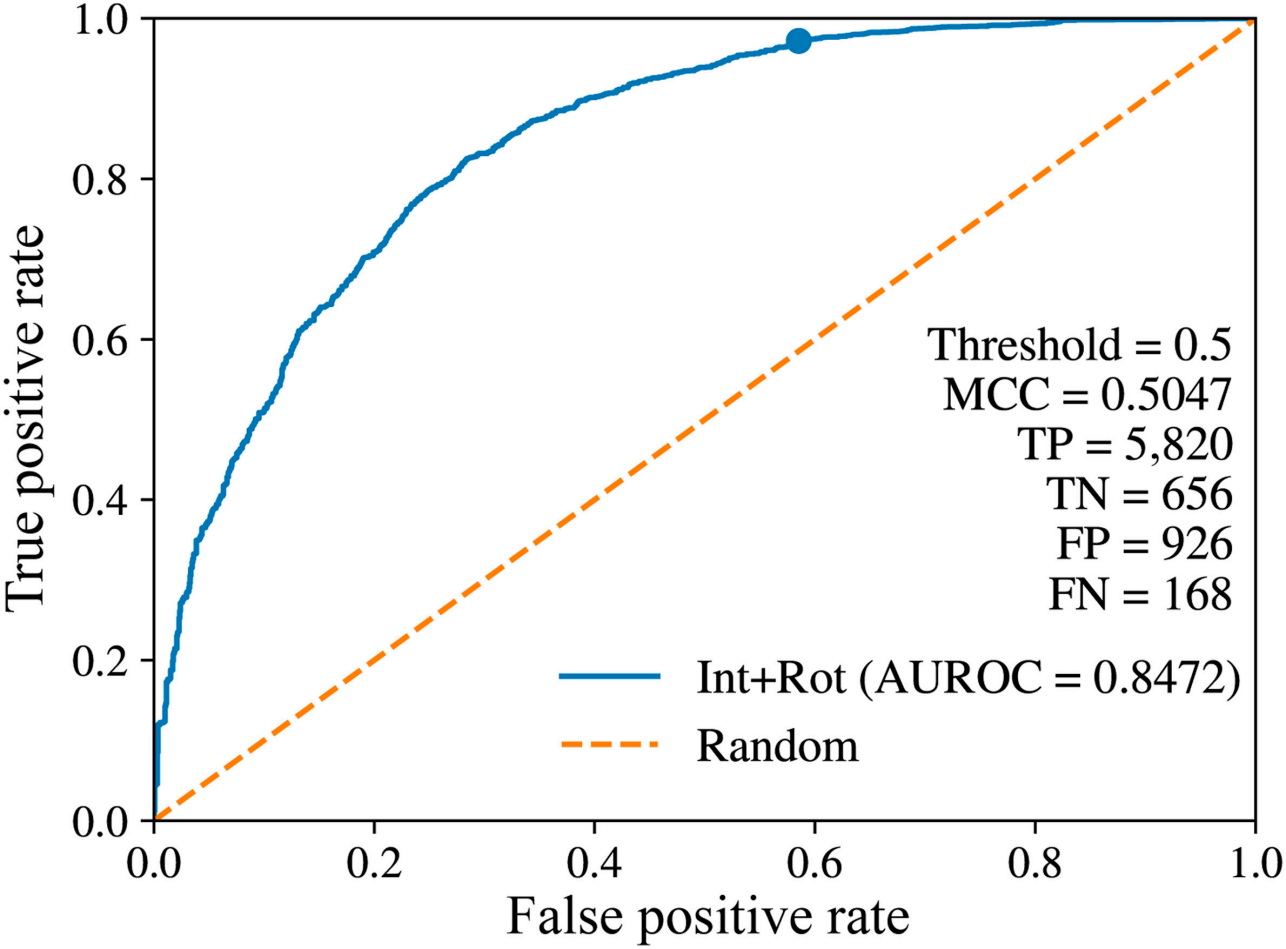
AUROC curve for the test set of the machine learning model built using interaction descriptors and rotamer energy (Int+Rot). The dashed line represents random classification performance. Indicated point corresponds to the threshold of 0.5.

When only interaction descriptors were used as features, the machine learning model achieved relatively low MCC and accuracy values of 0.336 and 0.816, respectively (Table 4). This may be because the interaction descriptors primarily capture favorable interactions but do not directly represent unfavorable side-chain conformations. To penalize unfavorable side-chain structures, we reconstructed the model with a rotamer-energy term—representing the probability of a given side-chain rotamer—as an additional descriptor. The resulting model—combining interaction descriptors and rotamer energy—exhibited improved performance, with MCC and accuracy increasing to 0.505 and 0.855, respectively. When the model was constructed using only the rotamer-energy term, the MCC decreased to 0.122. The confusion matrices for each analysis are provided in Table S6A to C. The area under the receiver operating characteristic (AUROC) curve and area under the precision–recall curve (AUPRC) for the model combining interaction descriptors and the rotamer-energy term are shown in Fig. 7 and Fig. S3, respectively.

**Table 4.** Performance comparison of machine learning models using different combinations of descriptor sets. Balanced accuracy was calculated as the arithmetic mean of sensitivity and specificity.

Descriptors	MCC	Accuracy	Precision	Balanced accuracy	AUROC	AUPRC
Int	0.336	0.816	0.620	0.620	0.807	0.932
Int+Rot	0.505	0.855	0.863	0.693	0.847	0.947
Rot	0.122	0.794	0.798	0.520	0.543	0.900

Int, interaction descriptors; Rot, rotamer energy; MCC, Matthews correlation coefficient; AUROC, area under the receiver operating characteristic; AUPRC, area under the precision–recall curve

Thus, the interaction descriptors defined in intDesc-AbMut, which are based on specified interaction criteria, capture relevant structural features and contain information for assessing side-chain crystal-structure-likeness. Note that the machine learning model was designed only to classify “crystal-structure-like”, not to directly evaluate improvements in binding affinity. Therefore, this model should not be interpreted as a binding affinity prediction model, but rather as a means of assessing the informativeness of descriptors.

### Classification thresholds and their impact on model performance

For classification of crystal-structure-likeness, side-chain conformations were labeled as “crystal-structure-like” if their RMSD relative to the corresponding crystal structure was ≤1.0 Å, and “non-crystal-structure-like” otherwise. To evaluate the robustness of this RMSD threshold, model performance was further assessed using alternative RMSD thresholds of 1.2 and 0.8 Å on the same dataset. We redefined the labels according to each threshold then performed hyperparameter grid search (Table S5B), trained the model, and applied the model to the test set. When early stopping was applied during model training, we observed premature termination of training (at 100 steps), indicating insufficient learning. Therefore, the number of training steps was fixed at 695, corresponding to the step at which early stopping occurred in the RMSD = 1.0 Å model. The model using an RMSD threshold of 1.0 Å achieved the highest MCC (Table 5 and Table S6D and E) and was therefore used for subsequent analyses.

**Table 5.** Model performance according to different RMSD thresholds for determining crystal-structure-likeness

RMSD threshold Å	MCC	AUROC	AUPRC
0.8	0.471	0.822	0.913
1.0	0.505	0.853	0.949
1.2	0.497	0.856	0.964

RMSD, root mean square deviation; MCC, Matthews correlation coefficient; AUROC, area under the receiver operating characteristic; AUPRC, area under the precision–recall curve

### Contribution of each descriptor to side-chain structure discrimination

**Fig. 8.**
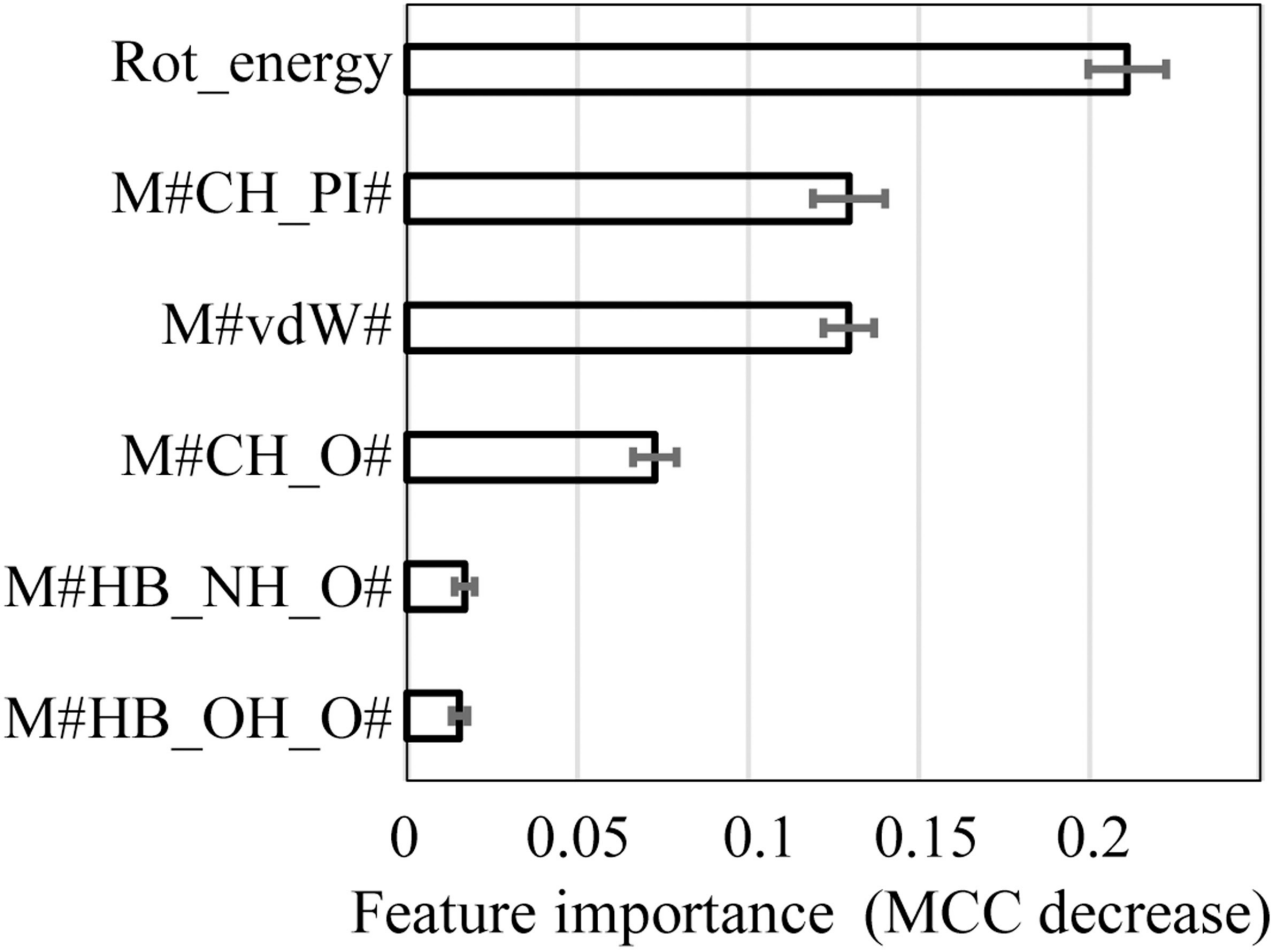
Importance of each descriptor to the predictive accuracy of the machine learning model. Error bars represent standard deviations calculated from 100-time repeated evaluations. Only the 6 top contributing descriptors are listed; the complete list is shown in Table S4.

The permutation importance method [33] was employed to determine the contribution of each descriptor to the predictive accuracy of the machine learning model (Fig. 8). The principal descriptors for determining the crystal-structure-likeness of a side-chain conformation were the rotamer energy (Rot_energy), CH···π interactions (M#CH_PI#), vdW interactions (M#vdW#), and CH···O interactions (M#CH_O#). Hydrogen bonds (M#HB_NH_O and M#HB_OH_O) were also important, albeit to a lesser extent.

The rotamer-energy term reflects the occurrence probability of side-chain rotamers; lower-probability conformations are penalized, which influences similarity to crystallographic structures. CH···π interactions represent weak, dispersion-driven hydrogen bonds between the aromatic ring and neighboring C–H group. For example, aromatic amino acids such as Phe, Tyr, Trp, and His tend to maximize CH···π interactions in “crystal-structure-like” models, whereas incorrectly modeled conformations result in poorly formed CH···π interactions.

In our definitions, vdW interactions comprise those between neighboring heavy atoms not assigned to other interaction types. Thus, vdW interactions exclude electrostatically favorable interactions—including weak hydrogen bonds—and favorable dipole–dipole interactions. Hence, they primarily reflect steric shape complementarity and packing between the mutant residue and its environment. Therefore, superior geometric complementarity should promote a more crystal-structure-like side-chain conformation.

CH···O interactions, which are also weak hydrogen bonds, contribute significantly to crystal-structure-like side-chain conformations, as proteins are rich in oxygen atoms—including backbone carbonyl oxygens and those in the side chains of Ser, Thr, Asn, Gln, Asp, and Glu—and donor hydrogens, such as backbone C_α_–H and side-chain C–H, CH₂, and CH₃ groups. The high polarity of oxygen atoms enables their involvement in strong (NH···O and OH···O) and weak (CH···O) hydrogen bonds, thereby stabilizing crystal structures.

Notably, CH···π and CH···O interactions, as weak hydrogen bonds, play substantial roles in determining the crystal-structure-likeness of side chains, emphasizing the value of intDesc-AbMut for visual inspection and understanding of interactions.

As defined in Table S1, the presence of each interaction depends on whether the distance between interacting heavy atoms is less than or equal to the sum of their vdW radii plus an additional 1 Å buffer. In intDesc-AbMut, this buffer value is implemented as an adjustable parameter and set to a default value of 1 Å for all interaction types. To assess the validity of this default buffer, we modified the buffer value to 0.8 and 1.2 Å for CH···π, vdW, and CH···O interactions, which showed particular importance in the above analysis. The resulting model performance metrics are summarized in Table S7, and the corresponding changes in the number of detected interactions are shown in Table S8. Model performance improved slightly in some cases; for example, MCC = 0.509 for a vdW interaction buffer of 0.8 Å, and MCC = 0.506 for a CH···O interaction buffer of 1.2 Å. However, overall, model performance was robust to changes in the buffer value, suggesting that the default setting of 1 Å for all interactions is reasonable. When constructing other machine learning models using interaction descriptors, the distance buffer used for interaction detection should be considered a tunable parameter for further performance optimization.

### Water-related interaction descriptors

As described in the “Generation and aggregation of interaction descriptors” section, we grouped interactions mediated by water molecules between the mutated residue and surrounding molecules (e.g., antigen or antibody), as well as direct interactions between water molecules and the mutated residue, to avoid an excessive increase in the number of descriptors. Specifically, these interactions were represented as counts of 2 descriptor types: “water-mediated interactions of the mutated residue (M##S##)” and “water–mutant interactions (M##S).” To evaluate whether this grouping strategy was appropriate, we also examined models in which water-related descriptors were treated separately in greater detail—specifically, 11 types of M#X#S, 11 types of M#X#S#X#, and 55 types of M#X#S#Y#. The results are summarized in Table 6, and the AUROC and AUPRC are presented in Fig. S4.

**Table 6.** Performance comparison between models using grouped and separately treated descriptors. Grouped descriptors: M##S, M##S##, M#X# (28), rot energy; individual descriptors: M#X#S (11), M#X#S#X# (11), M#X#S#Y# (55), M#X# (28), rot energy

Model	MCC	AUROC	AUPRC
Grouped	0.505	0.847	0.947
Individual	0.469	0.853	0.949

MCC, Matthews correlation coefficient; AUROC, area under the receiver operating characteristic; AUPRC, area under the precision–recall curve

The model using grouped water-related interaction descriptors showed a slightly higher MCC than the model in which water-related interactions were treated separately, whereas AUROC and AUPRC values were nearly identical between the 2 approaches. Therefore, the grouped model achieved comparable discriminative performance with fewer descriptors.

### Rotamer energy assumption

For the sake of methodological simplicity and computational tractability, we calculated the rotamer energy by assuming independent rotamer probabilities for 2 residues. However, previous studies have indicated that rotamer distributions can be influenced by local sequences and structural context. Taghizadeh et al. [44] and Dicks and Wales [45] reported that rotamer populations depend on adjacent residues in the primary sequence (*i* − 1 and *i* + 1), suggesting context-dependent modulation of rotamer probabilities. By quantifying statistically significant correlations between side-chain rotamer states arising from steric, solvation, and hydrogen-bond interactions, DuBay et al. [46] demonstrated that rotamer states are not always statistically independent across residues. Furthermore, Chamberlain and Bowie [47] showed that rotamer distributions differ between membrane and soluble proteins, indicating that rotamer preferences are sensitive to the surrounding physicochemical environment. In summary, rotamer probabilities may exhibit context dependence and limited transferability across structural environments. Therefore, the assumption of independence between the rotamer probabilities of 2 residues represents a simplifying approximation in our study. Incorporating the effects of sequence-adjacent residues and/or spatially neighboring residues into the rotamer-energy descriptor may further improve model performance.

### Contribution of each descriptor to individual side-chain conformations

**Fig. 9.**
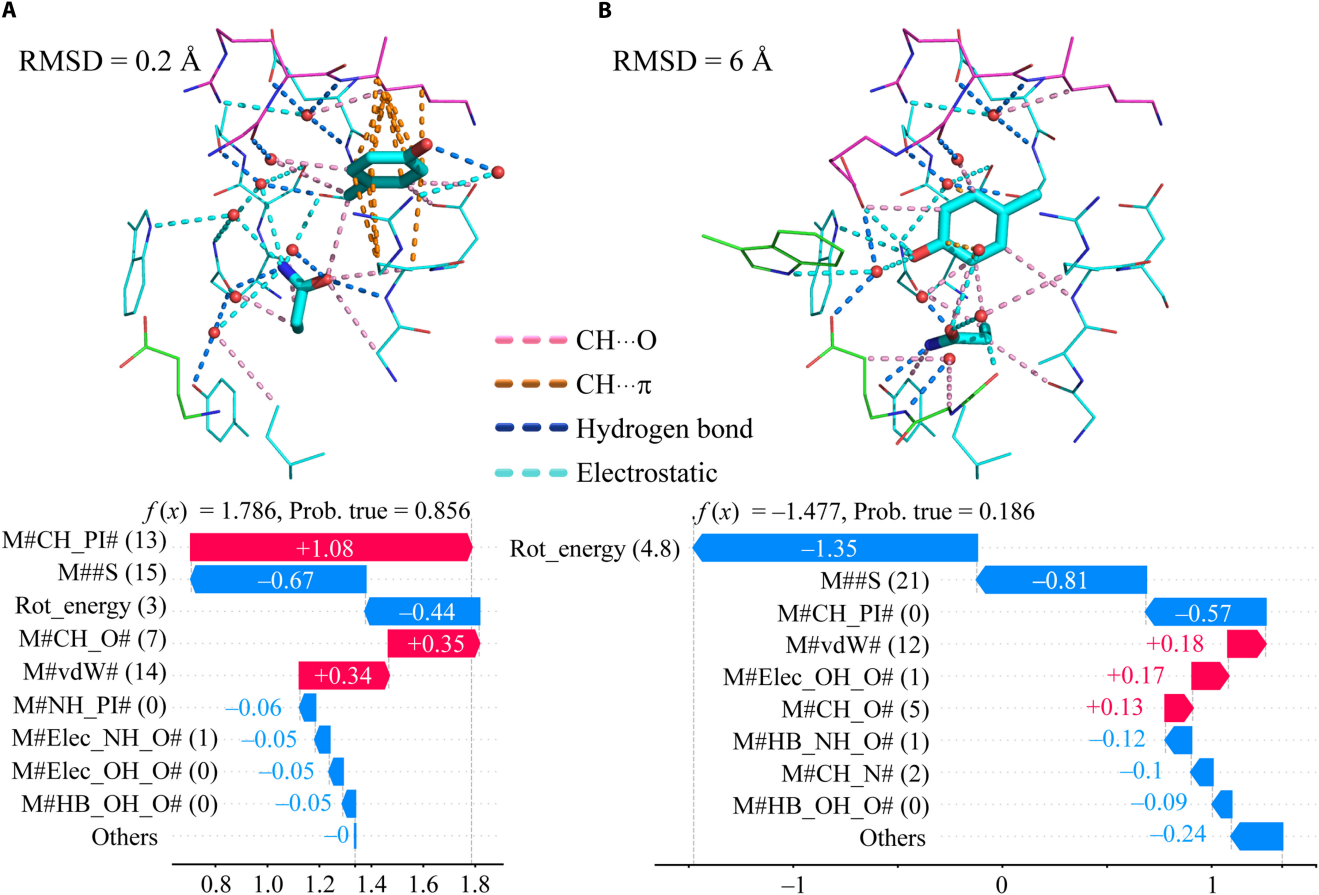
Contribution of interaction descriptors to the prediction value in the side-chain models. (A) Modeled structure correctly predicted as “crystal-structure-like”. (B) Modeled structure correctly predicted as “non-crystal-structure-like”. Tyr33 and Gln35 from PDB ID: 6VJN [48] were used. Cyan, antibody light chain; green, antibody heavy chain; magenta, antigen. van der Waals interactions are not shown. Hydrogen atoms are not shown. Only the side-chain structures of Y33 and Q35 are shown (sticks). In the water molecules, only oxygen atoms are shown (red spheres). The contribution of each feature to the probability of crystal-structure-likeness (Prob. true) was calculated using SHapley Additive exPlanations values [34], indicated as *f*(*x*).

Using the trained machine learning model, we present 2 examples of side-chain models for the residue pair Tyr33 and Gln35 (DPM) in the antibody light chain from the test dataset (6VJN [48]) to illustrate which descriptors contribute, and to what extent, to the determination of side-chain crystal-structure-likeness in individual predictions (Fig. 9). The machine learning model combining interaction descriptors and rotamer energy successfully distinguished the correct side-chain structure (Fig. 9A) from the incorrect side-chain structure (Fig. 9B). The probability of the “correct” modeled structure being “crystal-structure-like” was 0.856, with a side-chain RMSD of 0.2 Å relative to the crystal structure, whereas the probability of the “incorrect” modeled structure being “crystal-structure-like” was 0.186, with a significantly higher RMSD of 6 Å.

SHapley Additive exPlanations values for the case correctly predicted as “crystal-structure-like” showed that weak hydrogen bonds, specifically CH···π and CH···O interactions, made the greatest positive contribution to the classification results, followed by vdW interactions, reflecting steric shape complementarity (Fig. 9A). In contrast, for the case predicted as “non-crystal-structure-like”, the positive contributors were vdW, electrostatic OH···O interactions, and CH···O interactions, all of which exerted small effects (Fig. 9B). The strongest negative contributors to the “crystal-structure-like” classification results were the rotamer-energy term, water–side-chain interactions, and CH···π interactions.

### Implications for mutation analysis in antigen–antibody complexes

The residue-centric and descriptor-ready framework implemented in intDesc-AbMut provides a systematic approach for analyzing mutation-induced interaction changes in antigen–antibody complexes. By explicitly defining weak and noncanonical interaction categories, this tool can consistently identify interaction patterns that may otherwise be overlooked.

Although the aim of this study was not to predict binding affinity or automate mutation selection, the ability of intDesc-AbMut to quantitatively characterize local interaction environments offers a structured basis for interpreting the structural consequences of mutations. In this sense, intDesc-AbMut supports the transparent and reproducible structural rationalization of antibody mutations and provides a foundation for future supervised learning approaches incorporating experimentally measured affinity data.

## Conclusion

In this study, we present intDesc-AbMut, a residue-centric interaction analysis tool that systematically extracts, classifies, and visualizes diverse interaction types—including weak hydrogen bonds, sulfur-involving contacts, and orthogonal multipolar interactions—from antigen–antibody complex structures. By automating interaction identification previously performed manually, this software enables reproducible and transparent structural analysis of mutation-induced interaction changes.

Beyond visualization, intDesc-AbMut converts extracted interactions into quantitative descriptors that summarize the local interaction environment of designated residues. Through proof-of-concept machine learning analysis, we demonstrated that these descriptors contain structural information relevant to distinguishing “crystal-structure-like” from “non-crystal-structure-like” side-chain conformations. Note that this classifier was designed to evaluate descriptor informativeness with respect to structural plausibility, not to predict binding affinity.

Although we did not establish a direct relationship between interaction descriptors and experimental affinity improvement, our framework provides a systematic basis for characterizing mutation-induced interaction patterns. At present, the number of experimentally validated DPM cases remains limited; therefore, large-scale affinity-labeled datasets are required to rigorously evaluate the predictive utility of descriptor-based models for affinity optimization. Moreover, the proposed machine learning model assumes a fixed backbone geometry and independent rotamer probabilities; therefore, its applicability is limited to side-chain conformational assessment within antigen–antibody complexes of similar structural context. Future work should incorporate backbone flexibility, context-dependent rotamer coupling, and experimentally measured affinity data to improve model performance.

The intDesc-AbMut framework of interaction extraction and descriptor is not restricted to antibody engineering and may be extended to other protein–protein or protein–ligand systems. Moreover, integration with dynamic structural data, such as molecular dynamics trajectories, may enable time-resolved characterization of mutation-induced interaction changes.

Overall, intDesc-AbMut provides a structured and extendable framework for systematic residue-level interaction analysis that supports the mechanistic interpretation of antibody mutations, providing a methodological foundation for future affinity-aware modeling approaches.

## Data Availability

The descriptor calculation software intDesc-AbMut is provided at https://github.com/riken-yokohama-AI-drug/intDesc/tree/intDesc-AbMut (the GitHub release tag is v0.3.0). The dictionary-based file format converter, pdb2mol2.py, is provided at https://github.com/riken-yokohama-AI-drug/pdb2mol2 (v1.0.0). The rotamer energy calculator, rotamer_frequency.py, is provided at https://github.com/riken-yokohama-AI-drug/rotamer_frequency (v1.0.0). Examples for software execution are provided in the github pages. Training and test datasets (CSV format), including both the versions with integrated and nonintegrated water-mediated interactions, are available from the same GitHub repository.
