# Supplementary material for: intDesc-AbMut: A Tool for Describing and Understanding How Antibody Mutations Impact Their Environmental Interactions
Source: Comput Struct Biotechnol J. 2026 Apr 27;35(1):0027. doi: 10.34133/csbj.0027 (PMC13112784; doi:10.34133/csbj.0027)
Supplement: Supplementary 1 — Figs. S1 to S4 Tables S1 to S8 [file csbj.0027.f1.docx]

**intDesc-AbMut: a tool for describing and understanding how antibody mutations impact their environmental interactions**

Shuntaro Chiba^a†^, Masateru Ohta^a†^*, Tsutomu Yamane^a,b^, Yasushi Okuno^a,c^, Mitsunori Ikeguchi^a,b^

^a^ HPC- and AI-driven Drug Development Platform Division, RIKEN Center for Computational Science, Yokohama 230-0045, Japan

^b^ Graduate School of Medicinal Life Science, Yokohama City University, 230-0045, Yokohama, Japan

^c^ Department of Biomedical Data Intelligence, Graduate School of Medicine, Kyoto University, Kyoto 606-8507, Japan

^†^ Co-first authors

***Corresponding author:** [masateru.ota@a.riken.jp](mailto:masateru.ota@a.riken.jp)

**Table S1**. Definitions of interactions^a^

| Inter-action | Label | Don-or | D | A | Necessary conditions | Interaction scheme | Comment |
| --- | --- | --- | --- | --- | --- | --- | --- |
| Hydrogen bond (HB) | HB OH_O  HB NH_O  HB OH_N  HB NH_N | OH  NH  OH  NH | O  N  O  N | O  O  N  N | Dist. d(D:A) ≤ 3.2 Å  Ang. 0° ≤ ∠(H:D:A) ≤ 60°  Ang. 90° ≤ ∠(H:A:An1) ≤ 180°  Ang. 60° ≤ ∠(H:A:An2) ≤ 180° | 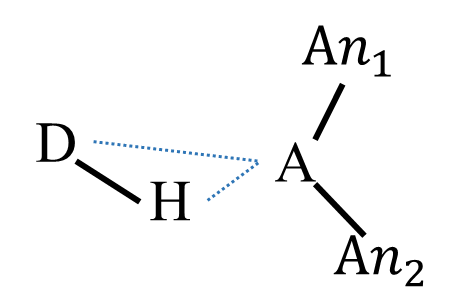 | An_2_ is optional and can be a hydrogen |
| Electro-static | Elec OH_O  Elec NH_O  Elec OH_N  Elec NH_N | OH  NH  OH  NH | O  N  O  N | O  O  N  N | Dist. 3.2 Å ≤ d(D:A) ≤ (RvdW(D) + RvdW(A) + 1.0 Å)  Ang. 0° ≤ ∠(H:D:A) ≤ 60°  Ang. 90° ≤ ∠(H:A:An1) ≤ 180°  Ang. 60° ≤ ∠(H:A:An2) ≤ 180° | 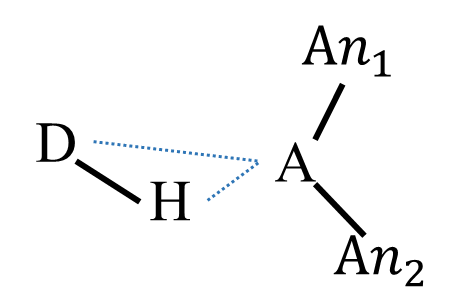 | An_2_ is optional and can be a hydrogen |
| CH···N | CH_N | CH | C | N | Dist. d(D:A) ≤ (RvdW(D) + RvdW(A) + 1.0 Å)  Dist. d(D:A) ≤ d(Dn1:A)  Dist. d(D:A) ≤ d(D:An1)  Dist. d(D:A) ≤ d(D:An2)  Dist. d(H:A) ≤ d(D:A)  ((Dist. d(H:A) ≤ 2.95 Å) OR (Dist. (2.95 Å < d(H:A) ≤ 3.10 Å) AND (Ang.135° ≤ ∠(D:H:A) ≤ 180°))) | 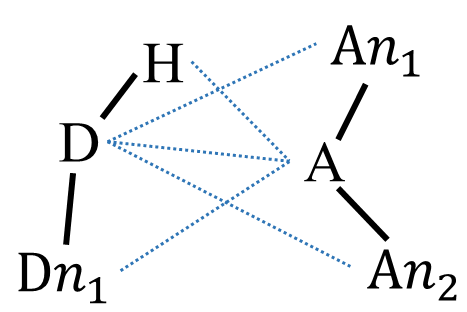 | An_2_ is optional and can be a hydrogen |
| CH···O | CH_O | CH | C | O | Dist. d(D:A) ≤ (RvdW(D) + RvdW(A) + 1.0 Å)  Dist. d(D:A) ≤ d(Dn1:A)  Dist. d(D:A) ≤ d(D:An1)  Dist. d(D:A) ≤ d(D:An2)  Dist. d(H:A) ≤ d(D:A)  Dist. d(H:A) ≤ 3.22 Å  Ang. 94.58° ≤ ∠(D:H:A) ≤ 180° | 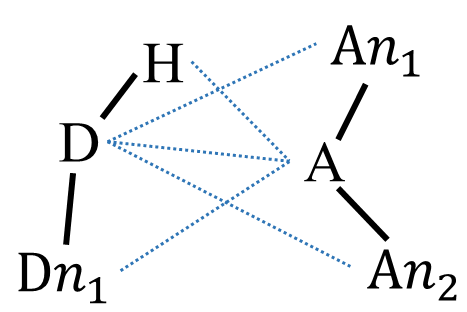 | An_2_ is optional and can be a hydrogen |
| CH···S | CH_S | CH | C | S | Dist. d(D:A) ≤ (RvdW(D) + RvdW(A) + 1.0 Å)  Dist. d(D:A) ≤ d(Dn1:A)  Dist. d(D:A) ≤ d(D:An1)  Dist. d(D:A) ≤ d(D:An2)  Dist. d(H:A) ≤ d(D:A)  ((Dist. d(H:A) ≤ 3.2 Å) OR (Dist. (3.2 Å < d(H:A) ≤ 3.3 Å) AND (Ang. 135° ≤ ∠(D:H:A) ≤ 180°))) | 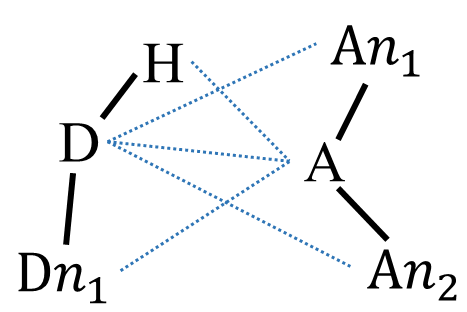 | An_2_ is optional and can be a hydrogen |
| OH···S | OH_S | OH | O | S | Dist. d(D:A) ≤ (RvdW(D) + RvdW(A) + 1.0 Å)  Dist. d(D:A) ≤ d(Dn1:A)  Dist. d(D:A) ≤ d(D:An1)  Dist. d(D:A) ≤ d(D:An2)  Dist. d(H:A) ≤ d(D:A)  Ang. 90° ≤ ∠(Dn1:D:A) ≤ 180° | 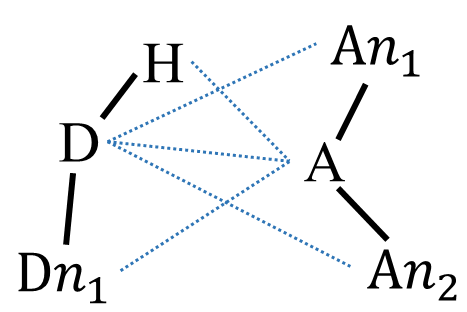 | An_2_ is optional and can be a hydrogen |
| NH···S | NH_S | NH | N | S | Dist. d(D:A) ≤ (RvdW(D) + RvdW(A) + 1.0 Å)  Dist. d(D:A) ≤ d(Dn1:A)  Dist. d(H:A) ≤ d(D:A)  Dist. d(D:A) ≤ d(D:An1)  Dist. d(D:A) ≤ d(D:An2)  (Dist. (d(H:A) ≤ 3.155Å) AND (Ang. 105° ≤ ∠(D:H:A) ≤ 180°)) OR (Dist. (3.155 Å< d(H:A) ≤ 3.4Å) AND (Ang. 165° ≤ ∠(D:H:A) ≤ 180°)) | 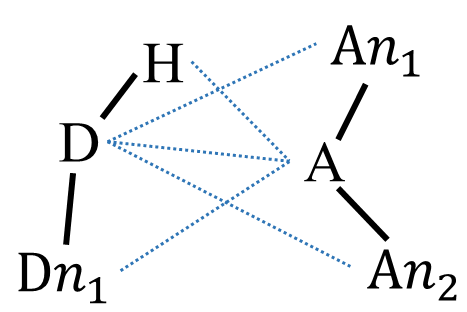 | An_2_ is optional and can be a hydrogen |
| SH···O | SH_O | SH | S | O | Dist. d(D:A) ≤ (RvdW(D) + RvdW(A) + 1.0 Å)  Dist. d(D:A) ≤ d(Dn1:A)  Dist. d(D:A) ≤ d(D:An1)  Dist. d(D:A) ≤ d(D:An2)  Dist. d(H:A) ≤ d(D:A) | 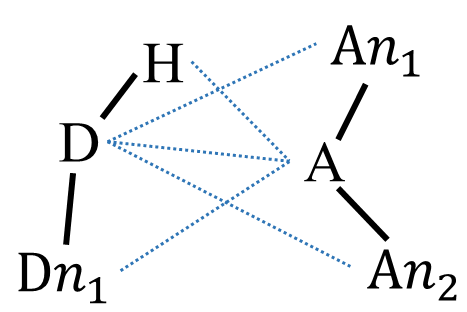 | An_2_ is optional and can be a hydrogen |
| SH···N | SH_N | SH | S | N | Dist. d(D:A) ≤ (RvdW(D) + RvdW(A) + 1.0 Å)  Dist. d(D:A) ≤ d(Dn1:A)  Dist. d(D:A) ≤ d(D:An1)  Dist. d(D:A) ≤ d(D:An2)  Dist. d(H:A) ≤ d(D:A) | 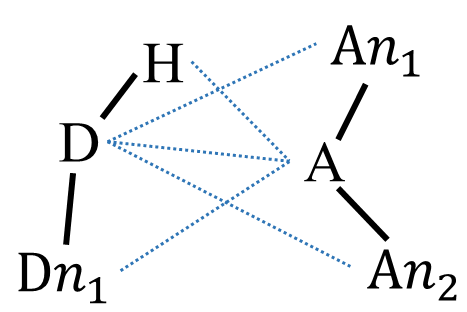 | An_2_ is optional and can be a hydrogen |
| SH···S | SH_S | SH | S | S | Dist. d(D:A) ≤ (RvdW(D) + RvdW(A) + 1.0 Å)  Dist. d(D:A) ≤ d(Dn1:A)  Dist. d(D:A) ≤ d(D:An1)  Dist. d(D:A) ≤ d(D:An2)  Dist. d(H:A) ≤ d(D:A)  Ang. 0° ≤ ∠(D:H:A) ≤ 90° | 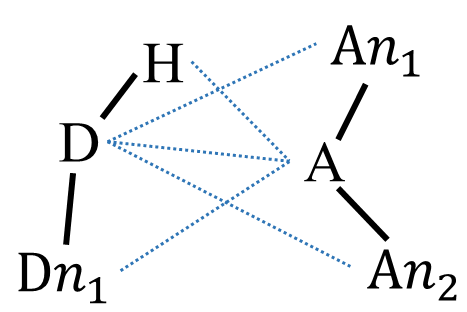 | An_2_ is optional and can be a hydrogen |
| $\pi-\pi$ stacking | PI_PI | Dist. d(Y:Z) ≤ (RvdW(Y) + RvdW(Z) + 1.0 Å)  θ1 ≤ 30° if 0° ≤ θ1 ≤ 90°  180 - θ1 ≤ 30° if 90° < θ1 ≤ 180° | | | | 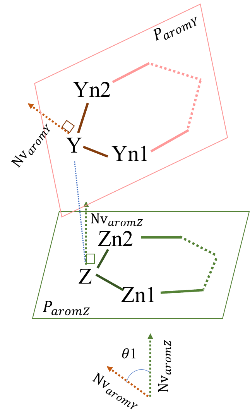 | Y, Yn1, Yn2, Z, Zn1, Zn2: aromatic;  NvaromP: the normal vector of plane P;  Angle θ1: the angle between NvaromY and NvaromZ |
| CH···π | CH_PI | CH | C | π | Dist. d(D:A) ≤ (RvdW(D) + RvdW(A) + 1.0 Å)  Dist. d(D:A) ≤ d(Dn1:A)  Dist. d(H:A) ≤ d(D:A)  Dist. d(Nrm:cn) ≤ d(cn:A)×1.4  (Dist. d(A:H) ≤ 3.195 Å) OR ((Dist. 3.195 Å<d(A:H) ≤ 3.325 Å) AND (Ang. 124.455° ≤ ∠(D:H:A) ≤ 180.0°)) | 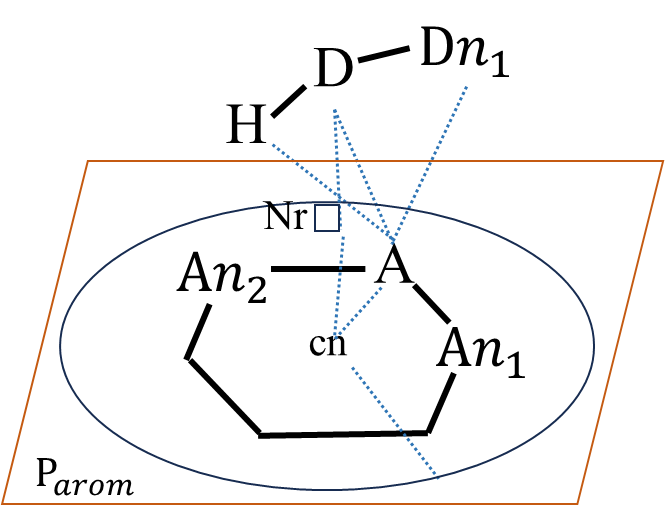 | A, $\mathrm{An}_{1}$, $\mathrm{An}_{2}$: Aromatic atoms |
| NH···π | NH_PI | NH | N | π | Dist. d(D:A) ≤ (RvdW(D) + RvdW(A) + 1.0 Å)  Dist. d(D:A) ≤ d(Dn1:A)  Dist. d(H:A) ≤ d(D:A)  Dist. d(Nrm:cn) ≤ d(cn:A) × 1.4  (Dist. d(A:H) ≤ 3.14Å) OR ((Dist. 3.14 Å < d(A:H) ≤ 3.365 Å) AND (Ang. 132.0° ≤ ∠(D:H:A) ≤ 180.0°)) | 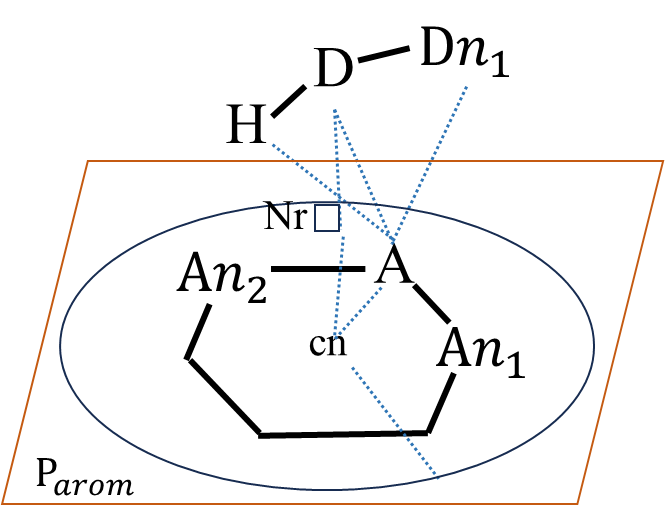 | A, $\mathrm{An}_{1}$, $\mathrm{An}_{2}$: Aromatic atoms |
| OH···$\pi$ | OH_PI | OH | O | $\pi$ | Dist. d(D:A) ≤ (RvdW(D) + RvdW(A) + 1.0 Å)  Dist. d(D:A) ≤ d(Dn1:A)  Dist. d(H:A) ≤ d(D:A)  Dist. d(Nr:cn) ≤ d(cn:A)×1.4  (Dist. d(A:H) ≤ 3.0 Å) OR ((Dist. 3.0 Å < d(A:H) ≤ 3.3Å) AND (Ang. 105.0° ≤ ∠(D:H:A) ≤ 180.0°)) | 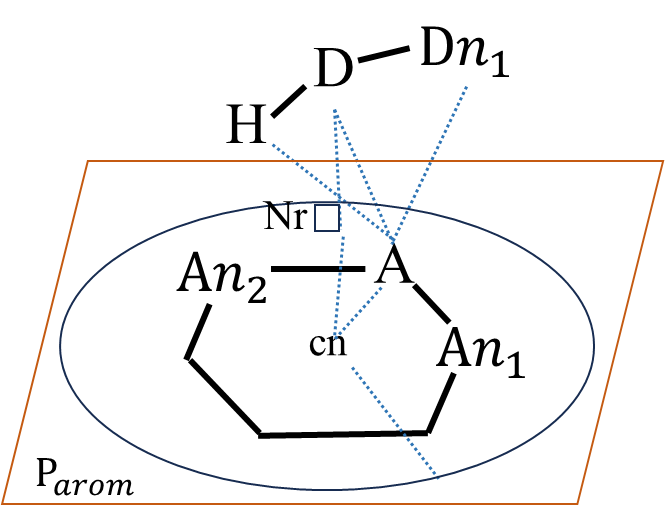 | A, $\mathrm{An}_{1}$, $\mathrm{An}_{2}$: Aromatic atoms |
| SH···$\pi$ | SH_PI | SH | S | $\pi$ | Dist. d(D:A) ≤ (RvdW(D) + RvdW(A) + 1.0 Å)  Dist. d(D:A) ≤ d(Dn1:A)  Dist. d(H:A) ≤ d(D:A)  Dist. d(Nr:cn) ≤ d(cn:A)×1.4  (Dist. d(A:H) ≤ 3.2 Å) OR ((Dist. 3.2 Å < d(A:H) ≤ 3.33 Å) AND (Ang. 120.0° ≤ ∠(D:H:A) ≤ 180.0°)) | 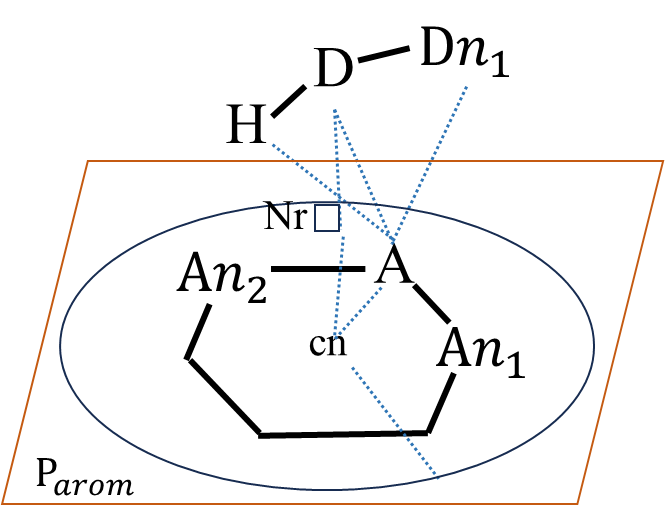 | A, $\mathrm{An}_{1}$, $\mathrm{An}_{2}$: Aromatic atoms |
| S···$\pi$ | S_PI | S | S | $\pi$ | Dist. d(D:A) ≤ (RvdW(D) + RvdW(A) + 1.0 Å)  Dist. d(D:A) ≤ d(Dn1:A)  Dist. d(D:A) ≤ d(Dn2:A)  Dist. d(Nr:cn) ≤ d(cn:A)×1.4  (Ang. 30° ≤ θ1 if θ1 ≤ 90°) OR  (Ang. 30° ≤ 180-θ1 if θ1 > 90°) | 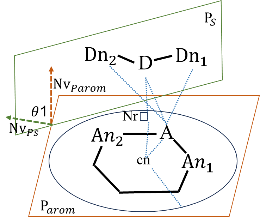 | S···$\pi$ is defined if the conditions on this line or the next line are satisfied |
| S···$\pi$ | S_PI | S | S | $\pi$ | Dist. d(D:A) ≤ (RvdW(D) + RvdW(A) + 1.0 Å)  Dist. d(D:A) ≤ d(Dn1:A)  Dist. d(Nr:cn) ≤ d(cn:A)×1.4  Dist. d(cn:D) ≤ d(cn:Dn1)  Ang. 120° ≤ ∠(Nr:D:Dn1) ≤ 180°  Dihedral angle 135° ≤ dihed(cn:Nr:D:Dn1) | 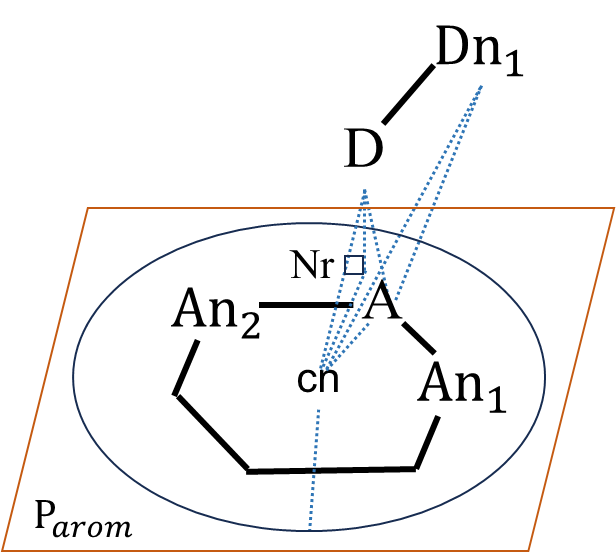 | S···$\pi$ is defined if the conditions on this line or the previous line are satisfied |
| S···O | S_O | S | S | O | Dist. d(D:A) ≤ RvdW(D) + RvdW(A) + 0.2 Å  Dist. d(D:A) ≤ d(Dn1:A)  Dist. d(D:A) ≤ d(Dn2:A)  Dist. d(D:A) ≤ d(D:An1)  Dist. d(D:A) ≤ d(D:An2) | 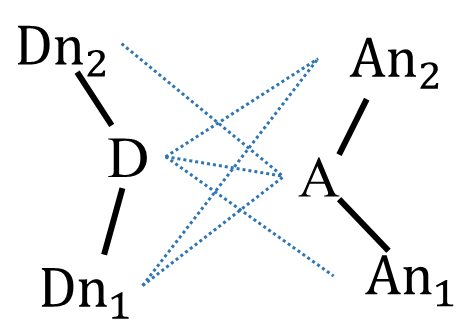 | Dn_2_ and An_2_ are optional and can be a hydrogen |
| S···N | S_N | S | S | N | Dist. d(D:A)≤ RvdW(D) + RvdW(A) + 0.2 Å  Dist. d(D:A) ≤ d(Dn1:A)  Dist. d(D:A) ≤ d(Dn2:A)  Dist. d(D:A) ≤ d(D:An1)  Dist. d(D:A) ≤ d(D:An2) | 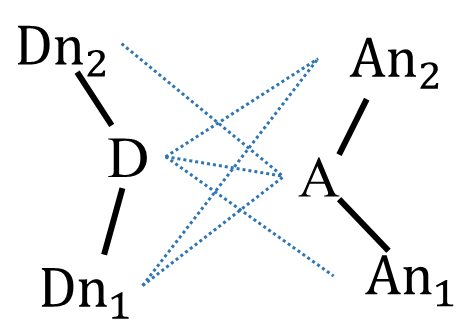 | Dn_2_ and An_2_ are optional and can be a hydrogen |
| S···S | S_S | S | S | S | Dist. d(D:A) ≤ RvdW(D) + RvdW(A) + 0.4 Å  Dist. d(D:A) ≤ d(Dn1:A)  Dist. d(D:A) ≤ d(Dn2:A)  Dist. d(D:A) ≤ d(D:An1)  Dist. d(D:A) ≤ d(D:An2) | 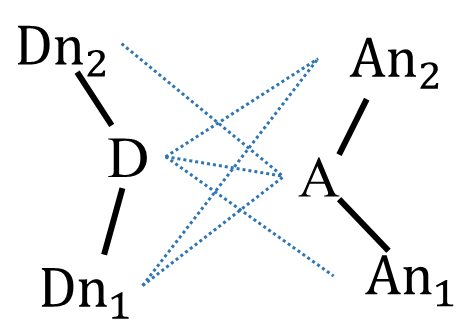 | Dn_2_ and An_2_ are optional and can be a hydrogen |
| Bond dipole | Dipo | \| $\delta^{+}$ − $\delta^{-}$ \| ≥ 0.2 in dipole 1  \| $\delta^{+}$ − $\delta^{-}$ \| ≥ 0.2 in dipole 2  Dist. d(Dp1+:Dp2-) ≤ (RvdW(Dp1+) + RvdW(Dp2-) + 1.0 Å)  Ang. 0° ≤ ∠(Dp1+:Dp2-:Dp2+) ≤ 90°  Ang. 0° ≤ ∠(Dp2-:Dp2+:Dp1-) ≤ 90°  Dist. d(cn1:cn2) ≤ (RvdW(Dp1+) + RvdW(Dp2-) + 1.0 Å)  165° ≤ θ1 ≤ 180° | | | | 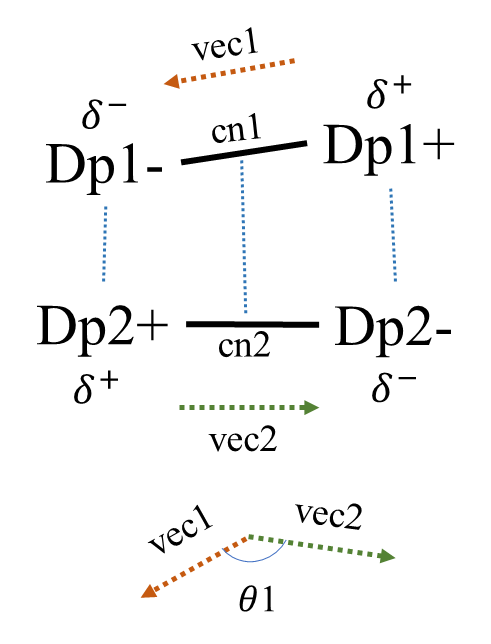 | cnX: the midpoint between DpX- and DpX+;  vecX: the vector from DpX+ to DpX-;  θ1: the angle between vec1 and vec2 |
| Orthogo-nal multi-polar interacti-on | OMulPol | \| $\delta^{+}$ − $\delta^{-}$ \| ≥ 0.2 in dipole 1  \| $\delta^{+}$ − $\delta^{-}$ \| ≥ 0.2 in dipole 2  Dist. d(Dp1-:Dp2+) ≤ RvdW(Dp1-) + RvdW(Dp2+) + 0.7Å  Dist. d(Dp1-:Dp2+) ≤ d(Dp1+: Dp2+)  Ang. 75°≤ ∠(Dp2-:Dp2+:Dp1-) ≤ 105°  Ang. 0° ≤ ∠(Nr:Dp1-:Dp2+) ≤ 35°  Ang. 150°≤ ∠(Nr:DP1-:Dp1+) ≤ 180° | | | | 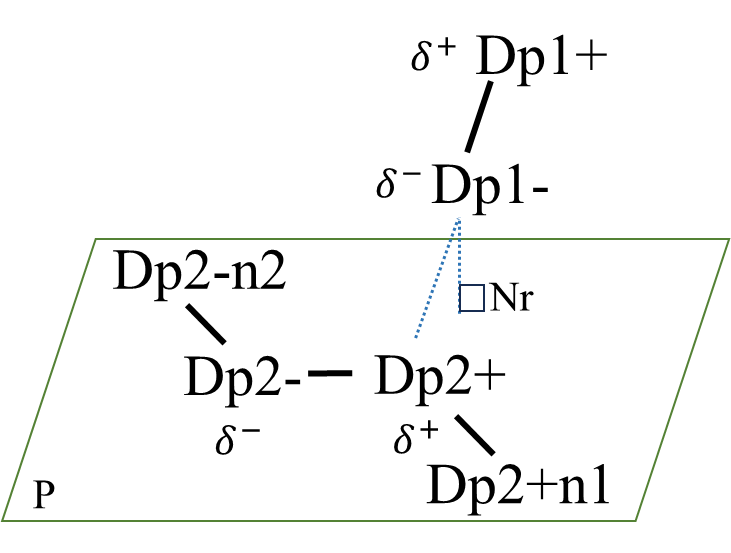 | Plane P is defined by Dp2-, Dp2+, and Dp2+n1 or  Dp2-n2 |
| Metal | Fe_A(element)  Zn_A(element)  Ca_A(element)  Mg_A(element)  Ni_A(element) | Fe  Zn  Ca  Mg  Ni | | A  A  A  A  A | Dist. d(D:A) ≤ (RvdW(D) + RvdW(A) + 0.2 Å)  Dist. d(D:A) ≤ d(D:An1)  Dist. d(D:A) ≤ d(D:An2) | 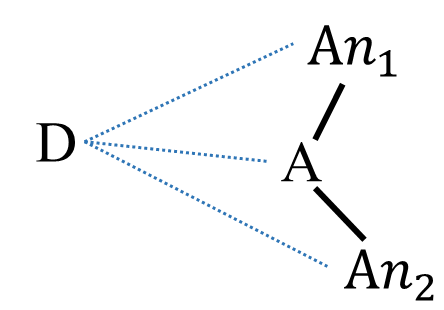 | An_2_ is optional;  A(element): the element of A |
| Ion | Na_A(element)  K_A(element)  Cl_D(element) | Na  K  D | | A  A  Cl | Dist. d(D:A) ≤ (RvdW(D) + RvdW(A) + 1.0 Å) | 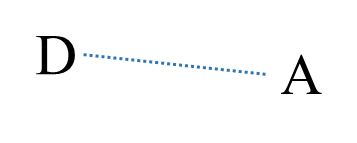 | A(element): the element of A |
| van der Waals | vdW | Dist. d(Y:Z) ≤ (RvdW(Y) + RvdW(Z) + 1.0 Å) | | | | 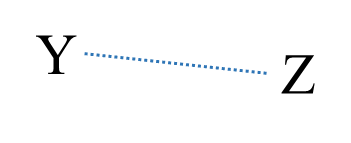 | Assigned if none of the interaction defined above |

^a^ Label: A prefix assigned to each interaction, used when displaying PyMOL menus and writing descriptors. D: Donor heavy atom, A: Acceptor heavy atom, H: Hydrogen. DnN, AnN, DpN, DpNn, Y, YnN, Z, ZnN: heavy atoms. RvdW(X): VDW radius of X (Å). Black lines: Any type of bond. Blue dotted lines: Distance, Dist.: Distance, Ang.: Angle. cn: the center of the aromatic ring. P: Plane. $P_{arom}$: the plane formed by aromatic atoms. Nr: the point where the perpendicular line from the atom to the plane P intersects with P. Nv: the normal vector of the plane. ${Nv}_{X}$: the normal vector of the plane X.θ1: the angle between two normal vectors Nv.

**Table S2**. Amino acids available in pdb2mol2.py

| Residue name^a^ | Note |
| --- | --- |
| GLY |  |
| ALA |  |
| VAL |  |
| LEU |  |
| ILE |  |
| PRO |  |
| PHE |  |
| TYR |  |
| TRP |  |
| SER |  |
| THR |  |
| HID | $N_{\delta}$-protonated His |
| HIE | $N_{\varepsilon}$-protonated His |
| HIP | $N_{\delta}$- and $N_{\varepsilon}$-protonated His |
| CYS |  |
| CYX | Cys with disulfide bond |
| MET |  |
| LYS |  |
| ARG |  |
| GLN |  |
| GLU |  |
| ASN |  |
| ASP |  |

^a^Residue names in C-terminal and N-terminal are prefixed by C and N, e.g., CGLY.

**Table S3**. PDB IDs used for training and test structure generation

| Training PDB (246 PDBs) |
| --- |
| (1)^a^ 5w0d, 6wg0, 5f9o, 5dtf, 5ds8, 5dub, 2fx7, 6a77, 1ors, 1i8k, 4jfx, 3fn0, 6gku, 1yqv, 5w3p, 6p7h, 6dcw, 5ngv, 7jmp, 6b5r, 6b5s, 1ce1, 2v17, 6meh, 6pdu, 4tsb, 4tsc, 5wn9, 6aq7, 1iqd, 3mnz, 3p0y, 3sob, 6w00, 6blh, 6mtt, 1f58, 5w5z, 2ypv, 3g5y, 5vpl, 5vpg, 3rvv, 3rvw, 6mvl, 3l5x, 4g6m, 2uzi, 4hha, 6uoe, 2vxq, 6k0y, 5xku, 4dgy, 6o24, 6ulf, 6vln, 5ggv, 6d0x, 6o26, 5ob5, 2cmr, 6yio, 2adf, 7kmi, 7neh, 5vkd, 5ucb, 5uek, 6s5a, 6tgg, 6fzr, 6fzq, 6frj, 5owp, 5a2l, 5a2k, 5a2j, 1sm3, 5a2i, 6k65, 5xcq, 5tkk, 5ea0, 6z2l, 3d85, 1e4w, 6uce, 5i8c, 6ucf, 2ih3, 2hvk, 1k4c, 1r3j, 5nph, 4h88, 4ma7, 5en2, 2fd6, 4al8, 4ala, 6bfs, 5kvg, 3mxw, 6uud, 1jps, 5yy4, 5e2v, 5e2w, 1fns, 1mvu, 1h0d, 6bzy, 4r3s, 4qxt, 4qy8, 6ddr, 5lqb, 5tl5, 6j5f, 6nyq, 4m1g, 1wej, 1kir, 1kiq, 1g7m, 1g7l, 1g7j, 1g7i, 1g7h, 1a2y, 1vfb, 6b5m, 6b5n, 5hdq, 5kve, 5kvf, 6mnq, 6b0g, 3bae, 3bkj, 3eys, 5eoq, 6pxr, 1pz5, 6kx1, 6pdr, 1qkz, 3v52, 3v4u, 3uo1, 3uyr, 5myk, 6vbo, 4tuk, 4tul, 6dc8, 4h0h, 6plh, 4ojf, 4lkx, 3ley, 6x8u, 1ndg, 1nby, 1nbz, 1dqj, 2eiz, 2eks, 1ua6, 1uac, 3a6c, 3a6b, 3a67, 2dqi, 2dqe, 2dqc, 1j1x, 1j1p, 1j1o, 3d9a, 2dqd, 2dqj, 4i77, 4gag; (2) 5j56, 6zrv, 6ir1, 5vak, 4kml, 4n9o, 4nbx, 4qo1, 6yu8, 5imk, 5iml, 4dka, 6qgw; (4) 2ny4, 2ny3, 2ny2, 2nxy, 2ny1; (5) 3idg, 3drq, 2f5b, 1tjg, 1u8i; (7) 3ffd, 2qhr, 6lra; (8) 4xmp, 1op9, 3eba; (10) 3se8, 4j6r; (11) 4xvs, 4xvt; (13) 5m14, 5m15; (14) 5u3n, 5u3o; (16) 1ri8; (17) 1zv5; (19) 2xwt; (20) 2xxm; (22) 3h0t; (23) 3se9; (25) 4hpy; (26) 4nzr; (28) 4wen; (29) 5e0q; (31) 5f21; (32) 5l21; (34) 5omm; (35) 5sy8; (37) 6app; (38) 6db6; (40) 6icc; (41) 6iea; (43) 6k3m; (44) 6pec; (46) 6rtw; (47) 6u55; (49) 6vjt; (50) 6x1w |
| Test PDB (28 PDBs) |
| (3) 3mlr, 3uji, 3ujj, 6b0s, 4z0x, 2b1h, 4hpo; (6) 3qsk, 2p49, 2p43, 2p44; (9) 3k74, 5m2j; (12) 5fcu, 4xvj; (15) 1osp; (18) 1zvy; (21) 3go1; (24) 4gft; (27) 4orz; (30) 5e8e; (33) 5l6y; (36) 5v6m; (39) 6i2g; (42) 6ir2; (45) 6rnk; (48) 6vjn; (51) 6xzu |

^a^CDR cluster group ID number is given in parentheses; a group comprises antibodies with CDR sequence identity values > 40%.

**Table S4**. Descriptor importance

| Descriptor name | Importance  (MCC decrease) | Standard deviation |
| --- | --- | --- |
| Rot_energy | 0.2110 | 0.0114 |
| M#CH_PI# | 0.1295 | 0.0106 |
| M#vdW# | 0.1294 | 0.0074 |
| M#CH_O# | 0.0726 | 0.0064 |
| M#HB_NH_O# | 0.0169 | 0.0029 |
| M#HB_OH_O# | 0.0154 | 0.0023 |
| M##S | 0.0148 | 0.0044 |
| M#Elec_OH_O# | 0.0086 | 0.0021 |
| M#PI_PI# | 0.0085 | 0.0025 |
| M#CH_N# | 0.0063 | 0.0019 |
| M##S## | 0.0051 | 0.0016 |
| M#NH_PI# | 0.0042 | 0.0032 |
| M#S_O# | 0.0011 | 0.0006 |
| M#Dipo# | 0.0011 | 0.0011 |
| M#Elec_OH_N# | 0.0007 | 0.0006 |
| M#Elec_NH_O# | 0.0005 | 0.0019 |
| M#OMulPol# | 0.0003 | 0.0009 |
| M#HB_NH_N# | 0.0001 | 0.0004 |
| M#OH_PI# | 0.0001 | 0.0003 |
| M#Elec_NH_N# | 0.0000 | 0.0000 |
| M#NH_S# | 0.0000 | 0.0000 |
| M#OH_S# | 0.0000 | 0.0000 |
| M#SH_N# | 0.0000 | 0.0000 |
| M#SH_O# | 0.0000 | 0.0000 |
| M#SH_PI# | 0.0000 | 0.0000 |
| M#SH_S# | 0.0000 | 0.0000 |
| M#S_N# | 0.0000 | 0.0000 |
| M#S_PI# | 0.0000 | 0.0000 |
| M#S_S# | 0.0000 | 0.0000 |
| M#HB_OH_N# | 0.0000 | 0.0001 |
| M#CH_S# | −0.0010 | 0.0012 |

**Table S5**. (a) Hyperparameters searched

| Hyperparameter | Name in XGBoost python package | Search range |
| --- | --- | --- |
| Max depth | max_depth | 2, 3, 4, 5, 6, 8, 10 |
| Minimum sum of instance weight needed in a child | min_child_weight | 1, 3, 5, …, 31 |
| Gamma | Gamma | 0.1, 0.2, …, 0.6 |
| Subsample ratio of columns when constructing each tree | colsample_bytree | 0.3, 0.4, …, 0.9, 1 |
| Constraints of the estimation of the weights for each decision tree | max_delta_step | 0.125, 0.25, 0.5, 1, 1.5, 2, 4, 0 (“0” means no constraints.) |

(b) Hyperparameters selected

| Label threshold | Model | | Best hyperparameter | | | | |
| --- | --- | --- | --- | --- | --- | --- | --- |
| RMSD Å | Interaction descriptor | Rotamer energy | max_depth | min_child_weight | Gamma | colsample_bytree | max_delta_step |
| 1 | + | − | 6 | 29 | 0.5 | 0.7 | 0  (No constraints) |
| 1 | + | + | 3 | 29 | 0.1 | 0.6 | 1 |
| 1 | − | + | 6 | 27 | 0.5 | 0.3 | 0.25 |
| 0.8 | + | + | 2 | 25 | 0.3 | 0.6 | 0.5 |
| 1.2 | + | + | 4 | 31 | 0.6 | 0.9 | 1 |

**Table S6**. (a) Predictive performance (interaction descriptors)

| Confusion matrix | | | | | | | |
| --- | --- | --- | --- | --- | --- | --- | --- |
|  | | | Ground truth | | | | |
|  |  |  | Crystal-structure-like (correct) | | | Non-crystal-structure-like (incorrect) | |
| Prediction | Crystal-structure-like (correct) | | 5,730 | | | 1,133 | |
|  | Non-crystal-structure-like (incorrect) | | 258 | | | 449 | |
| Total | | | 5,988 | | | 1,582 | |
|  | | | | | | | |
| Metric | | | | | | | |
| MCC | Accuracy | Precision | | Balanced accuracy | AUROC | | AUPRC |
| 0.336 | 0.816 | 0.835 | | 0.620 | 0.807 | | 0.932 |

(b) Predictive performance (Rot energy + interaction descriptors)

| Confusion matrix | | | | | | | |
| --- | --- | --- | --- | --- | --- | --- | --- |
|  | | | Ground truth | | | | |
|  |  |  | Crystal-structure-like (correct) | | | Non-crystal-structure-like (incorrect) | |
| Prediction | Crystal-structure-like (correct) | | 5,820 | | | 926 | |
|  | Non-crystal-structure-like (incorrect) | | 168 | | | 656 | |
| Total | | | 5,988 | | | 1,582 | |
|  | | | | | | | |
| Metric | | | | | | | |
| MCC | Accuracy | Precision | | Balanced accuracy | AUROC | | AUPRC |
| 0.505 | 0.855 | 0.863 | | 0.693 | 0.847 | | 0.947 |

(c) Predictive performance (Rot energy)

| Confusion matrix | | | | | | | |
| --- | --- | --- | --- | --- | --- | --- | --- |
|  | | | Ground truth | | | | |
|  |  |  | Crystal-structure-like (correct) | | | Non-crystal-structure-like (incorrect) | |
| Prediction | Crystal-structure-like (correct) | | 5,931 | | | 1,504 | |
|  | Non-crystal-structure-like (incorrect) | | 57 | | | 78 | |
| Total | | | 5,988 | | | 1,582 | |
|  | | | | | | | |
| Metric | | | | | | | |
| MCC | Accuracy | Precision | | Balanced accuracy | AUROC | | AUPRC |
| 0.122 | 0.794 | 0.798 | | 0.52 | 0.543 | | 0.900 |

(d) Predictive performance (RMSD = 0.8 Å, Rot energy + interaction descriptors)

| Confusion matrix | | | | | | | |
| --- | --- | --- | --- | --- | --- | --- | --- |
|  | | | Ground truth | | | | |
|  |  |  | Crystal-structure-like (correct) | | | Non-crystal-structure-like (incorrect) | |
| Prediction | Crystal-structure-like (correct) | | 5,377 | | | 1,214 | |
|  | Non-crystal-structure-like (incorrect) | | 193 | | | 786 | |
| Total | | | 5,570 | | | 2,000 | |
|  | | | | | | | |
| Metric | | | | | | | |
| MCC | Accuracy | Precision | | Balanced accuracy | AUROC | | AUPRC |
| 0.471 | 0.814 | 0.816 | | 0.679 | 0.822 | | 0.913 |

(e) Predictive performance (RMSD = 1.2 Å, Rot energy + interaction descriptors)

| Confusion matrix | | | | | | | |
| --- | --- | --- | --- | --- | --- | --- | --- |
|  | | | Ground truth | | | | |
|  |  |  | Crystal-structure-like (correct) | | | Non-crystal-structure-like (incorrect) | |
| Prediction | Crystal-structure-like (correct) | | 6,132 | | | 751 | |
|  | Non-crystal-structure-like (incorrect) | | 168 | | | 519 | |
| Total | | | 6,300 | | | 1,270 | |
|  | | | | | | | |
| Metric | | | | | | | |
| MCC | Accuracy | Precision | | Balanced accuracy | AUROC | | AUPRC |
| 0.497 | 0.879 | 0.891 | | 0.691 | 0.856 | | 0.964 |

**Table S7**. Sensitivity analysis of the definitions of interaction descriptors for predictive performance

|  | Buffer  Å | MCC | Accuracy | Precision | Balanced accuracy | AUROC | AUPRC | TP | TN | FP | FN |
| --- | --- | --- | --- | --- | --- | --- | --- | --- | --- | --- | --- |
| Baseline | 1 | 0.505 | 0.855 | 0.863 | 0.693 | 0.8470 | 0.9470 | 5,820 | 656 | 926 | 168 |
| CH_PI | 0.8 | 0.500 | 0.854 | 0.863 | 0.693 | 0.8472 | 0.9463 | 5,805 | 660 | 922 | 183 |
| CH_PI | 1.2 | 0.502 | 0.855 | 0.864 | 0.695 | 0.8475 | 0.9466 | 5,803 | 666 | 916 | 185 |
| vdW | 0.8 | 0.509 | 0.857 | 0.864 | 0.696 | 0.8485 | 0.9474 | 5,819 | 665 | 917 | 169 |
| vdW | 1.2 | 0.505 | 0.855 | 0.864 | 0.696 | 0.8485 | 0.9469 | 5,807 | 667 | 915 | 181 |
| CH_O | 0.8 | 0.500 | 0.854 | 0.861 | 0.690 | 0.8469 | 0.9465 | 5,823 | 645 | 937 | 165 |
| CH_O | 1.2 | 0.506 | 0.856 | 0.863 | 0.695 | 0.8476 | 0.9466 | 5,816 | 662 | 920 | 172 |

**Table S8**. **Change in the total number of interactions in the sensitivity analysis**

|  | Buffer  Å | M#CH_PI# | M#vdW | M#CH_O# | M#CH_N# | M##S | M#S# |
| --- | --- | --- | --- | --- | --- | --- | --- |
| Baseline^a^ | 1 | 477,644 | 1,091,366 | 442,855 | 98,318 | 456,633 | 2,005,467 |
| CH_PI | 0.8 | −14,976 | 0 | 0 | 164 | 0 | 0 |
| CH_PI | 1.2 | 564 | 0 | 0 | 0 | 0 | 0 |
| vdW | 0.8 | 0 | −76,238 | 0 | 0 | −9,597 | −77,786 |
| vdW | 1.2 | 0 | 33,873 | 0 | 0 | 5,385 | 49,207 |
| CH_O | 0.8 | 0 | 0 | −13,553 | 0 | −2,620 | −18,205 |
| CH_O | 1.2 | 0 | 0 | 21 | 0 | 0 | 0 |

^a^Baseline reports the total number of interactions before modifying the buffer length


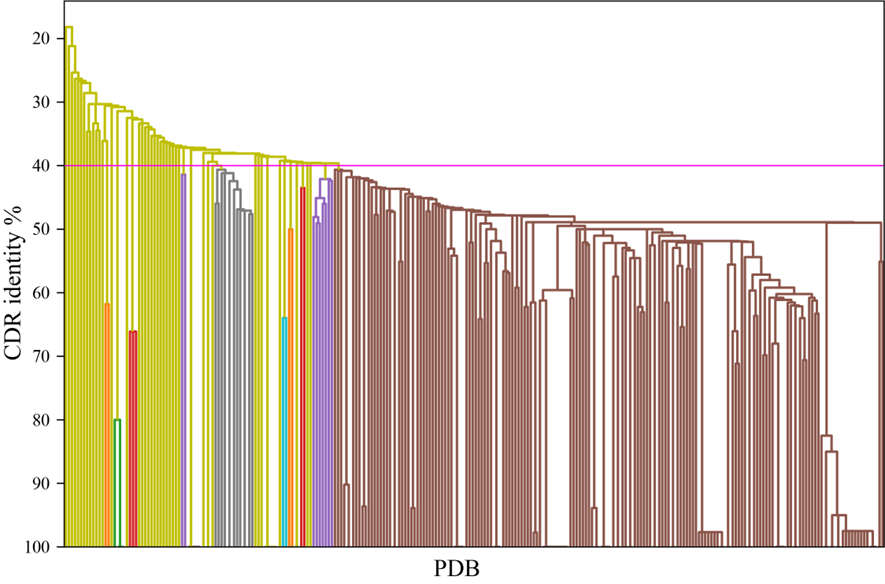


**Figure S1**. Clustering of PDB files used to derive training and test datasets. CDR identity defined in the main text was used for the single-linkage clustering implemented in SciPy 1.5.3 [1]. Horizontal magenta line represents the 40% threshold used to define different clusters.


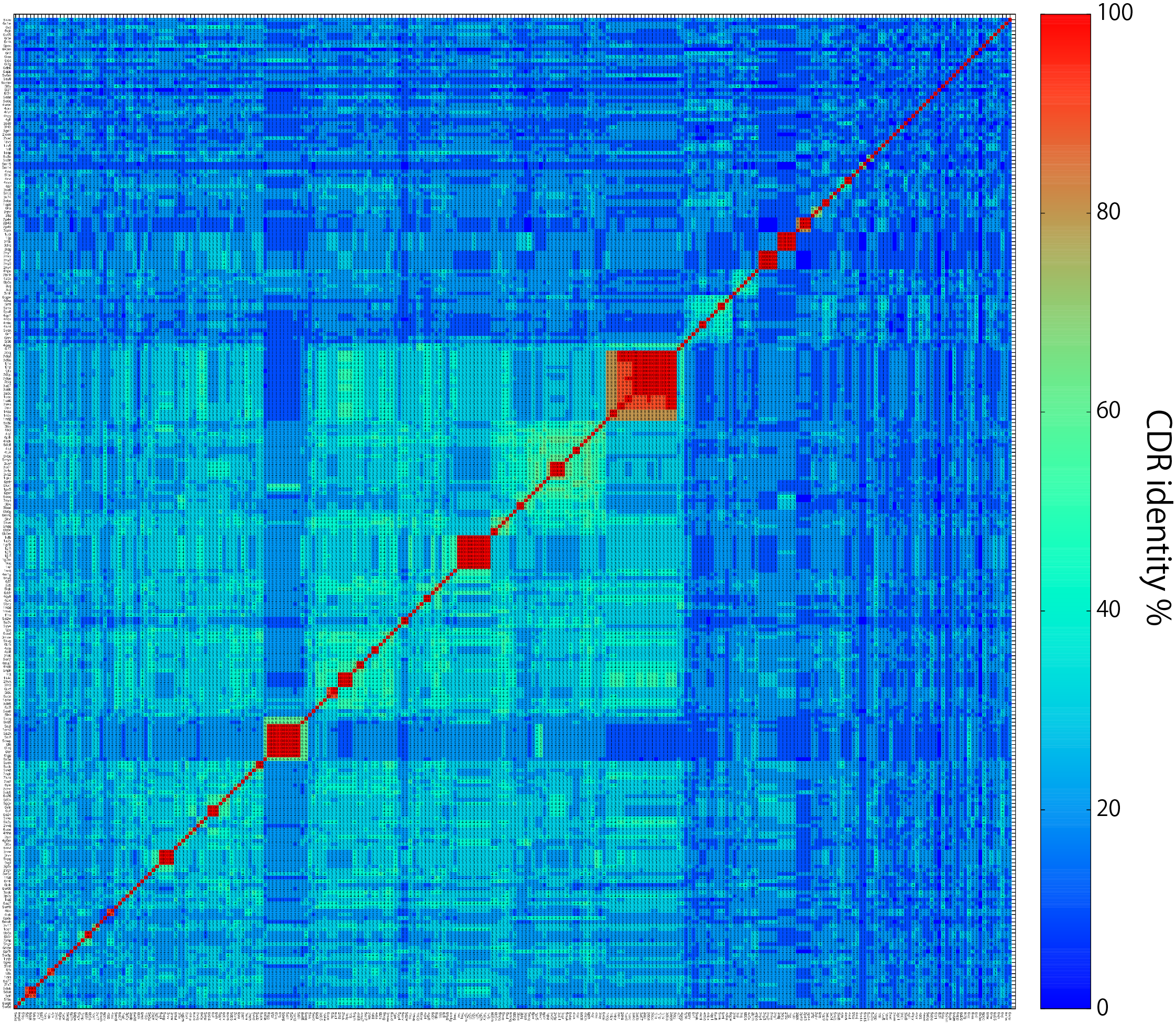


**Figure S2**. CDR identity of different PDBs sorted by cluster IDs (**Table S3**)


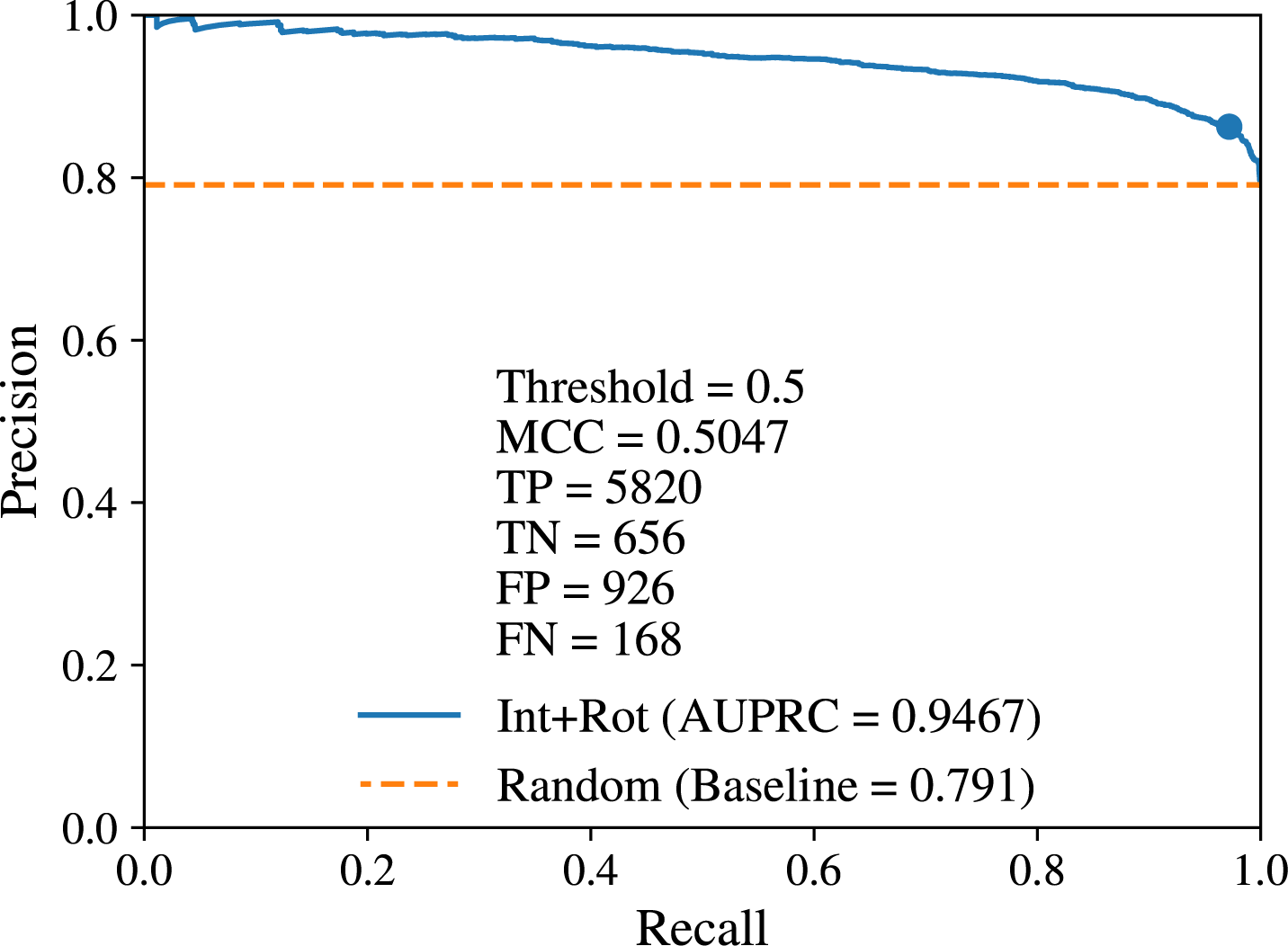


**Figure S3**. AUPRC curve of the test set for the machine learning model built using interaction descriptors and rotamer energy (Int+Rot). Dashed line represents random classification performance. Indicated point corresponds to a threshold of 0.5.

**
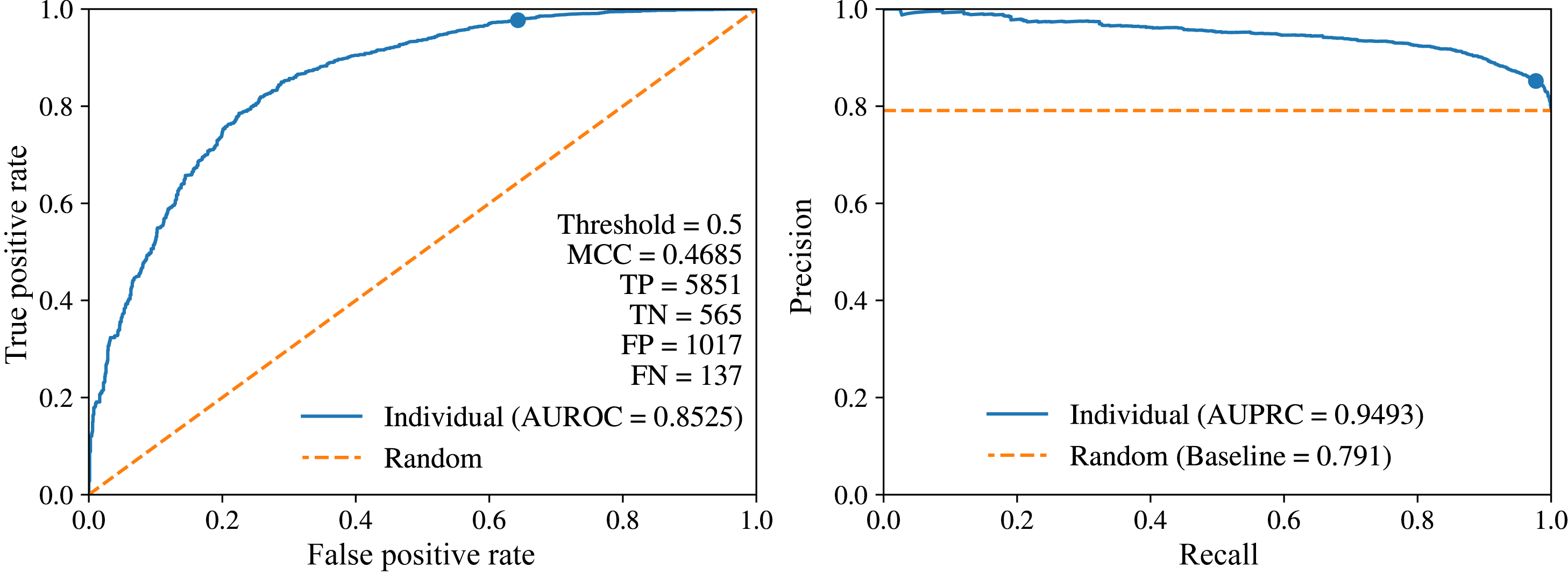
**

**Figure S4.** AUROC and AUPRC curves on the test set for the machine learning model integrating interaction descriptors and rotamer energy, in which water-related descriptors were treated separately in greater detail (Individual). Dashed lines represent random classification performance. Highlighted points correspond to a decision threshold of 0.5.

**References**

[1] Virtanen P, Gommers R, Oliphant TE, Haberland M, Reddy T, et al. (2020) SciPy 1.0: fundamental algorithms for scientific computing in Python. Nat Methods 17(3): 261-272.
